# Progressive cancer targeting by programmable aptamer‐tethered nanostructures

**DOI:** 10.1002/mco2.775

**Published:** 2024-10-20

**Authors:** Fatemeh Mohammadi, Hamed Zahraee, Farkhonde Zibadi, Zahra Khoshbin, Mohammad Ramezani, Mona Alibolandi, Khalil Abnous, Seyed Mohammad Taghdisi

**Affiliations:** ^1^ Targeted Drug Delivery Research Center Pharmaceutical Technology Institute Mashhad University of Medical Sciences Mashhad Iran; ^2^ Department of Pharmaceutical Biotechnology School of Pharmacy Mashhad University of Medical Sciences Mashhad Iran; ^3^ Department of Medical Biotechnology and Nanotechnology Faculty of Medicine Mashhad University of Medical Sciences Mashhad Iran; ^4^ Pharmaceutical Research Center Pharmaceutical Technology Institute Mashhad University of Medical Sciences Mashhad Iran; ^5^ Department of Medicinal Chemistry School of Pharmacy Mashhad University of Medical Sciences Mashhad Iran

**Keywords:** aptamer, bioimaging, cancer, DNA nanotechnology, gene therapy, nucleic acid nanostructures, targeted drug delivery

## Abstract

Scientific research in recent decades has affirmed an increase in cancer incidence as a cause of death globally. Cancer can be considered a plurality of various diseases rather than a single disease, which can be a multifaceted problem. Hence, cancer therapy techniques acquired more accelerated and urgent approvals compared to other therapeutic approaches. Radiotherapy, chemotherapy, immunotherapy, and surgery have been widely adopted as routine cancer treatment strategies to suppress disease progression and metastasis. These therapeutic approaches have lengthened the longevity of countless cancer patients. Nonetheless, some inherent limitations have restricted their application, including insignificant therapeutic efficacy, toxicity, negligible targeting, non‐specific distribution, and multidrug resistance. The development of therapeutic oligomer nanoconstructs with the advantages of chemical solid‐phase synthesis, programmable design, and precise adjustment is crucial for advancing smart targeted drug nanocarriers. This review focuses on the significance of the different aptamer‐assembled nanoconstructs as multifunctional nucleic acid oligomeric nanoskeletons in efficient drug delivery. We discuss recent advancements in the design and utilization of aptamer‐tethered nanostructures to enhance the efficacy of cancer treatment. Valuably, this comprehensive review highlights self‐assembled aptamers as the exceptionally intelligent nano‐biomaterials for targeted drug delivery based on their superior stability, high specificity, excellent recoverability, inherent biocompatibility, and versatile functions.

## Graphical abstract

Schematic of the drug delivery mechanism based on nanotrains and circular bivalent aptamer nanoskeleton carriers targeting the cancer cell.

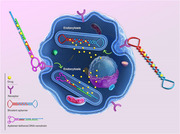

## 1 INTRODUCTION

Cancer is a heterogeneous disease in which genomic or molecular changes cause uncontrolled growth and proliferation of cells, a lack of differentiation between cells, and their successful invasion into different body parts.^1^ Cancer is the second leading cause of death worldwide, and the death rate has tripled (28%) over the same period in the last decade. Differences in mortality rates in different parts of the world are influenced by strategies for prevention, diagnosis, screening, and appropriate treatments.^2^ Currently, chemotherapy is one of the conventional cancer treatment approaches. Although chemotherapy has greatly helped to improve the treatment process, it still faces a serious challenge due to the lack of optimal drug access to the target cancer cells, limited bioavailability, drug resistance, need for high drug doses, and subsequent increase in systemic toxicity.^3^, ^4^ For this reason, prescribing the drug and ensuring its correct delivery without reducing its inherent effects or occurrence of its side effects is one of the main concerns of the medical field.^5^ Given the commitment to increase treatment efficacy for patient compliance and health promotion, a serious urgency is expected to develop innovative drug delivery systems, diagnostics, and targeted therapy. In the late 1800s, the concept of targeted therapy was first explored, but it quickly gained an important place in improving cancer treatment and reducing cytotoxicity.^6^, ^7^ Strategies based on targeted drug delivery systems can formulate and store various pharmaceutical agents. In this way, controlled concentration of drugs and access to them at a specific location in the body are accelerated without reducing their therapeutic effect, thereby maximizing therapeutic efficacy.^8^, ^9^, ^10^

DNA‐based nanostructures have emerged as the most promising candidates for safe drug delivery systems based on their acceptable physicochemical properties.^11^, ^12^ In terms of chemical properties, DNA plays a very important role due to the self‐assembly feature of molecules without the need for external experimental parameters.^13^ In the past few decades, single‐stranded DNA (ssDNA) and double‐stranded DNA (dsDNA) have been introduced as an inspiration for creating nanostructures with distinct sizes and shapes for targeted drug delivery to cells. Multifunctional DNA nanostructures offer unique possibilities such as effective cancer targeting, bioimaging, sensing, and drug delivery relying on their inherent properties, including precise control of size, shape, and function, steric stability, facile synthesis, biocompatibility, and biodegradability.^14^, ^15^ Because of the flexibility in formation, the desired DNA nanostructures can be designed according to the cargo required to load different therapeutic agents.^16^, ^17^ Aptamers can be easily incorporated into DNA nanostructures as DNA or RNA single strands with special three‐dimensional (3D) structures.^18^, ^19^, ^20^, ^21^ In this field, aptamers have attracted specific attention owing to their stability, high target affinity, easy chemical reform, low immunogenicity, and rapid tissue penetration.^22^, ^23^, ^24^, ^25^, ^26^, ^27^ Although some groups of aptamers can be used alone for disease treatment, they need a relatively high dose to be effective; while they are promising and more effective in the form of combined platforms with drugs.^28^

There have been numerous breakthroughs in the realm of diagnostics related to aptamer‒drug conjugates up until this point.^29^, ^30^, ^31^ Most of them use the covalent combination of drugs and aptamers for targeted drug delivery and release^32^, ^33^; unfortunately, the ratio of drug to carrier is limited in these assays.^34^ On the other hand, the aptamer‒nanomaterial complexes are not an ideal option for diagnostic purposes due to their complexity, limited stability, and uneconomical and laborious preparation.^35^, ^36^, ^37^, ^38^, ^39^ Therefore, simple, safe, cost‐effective, and biocompatible platforms with high therapeutic efficiency and maximum tolerable doses are still in demand. To address this challenge, researchers have advanced DNA aptamer‐tethered nanostructures as long linear dsDNA structures, which provide numerous addressable sites for loading therapeutic and diagnostic agents.^40^, ^41^, ^42^, ^43^ In this way, we will have a powerful tool to distinguish tumor cells from normal cells and subsequently minimize the negative effects of non‐specific uptake and reduce drug‐induced cytotoxicity.

**FIGURE 1:**
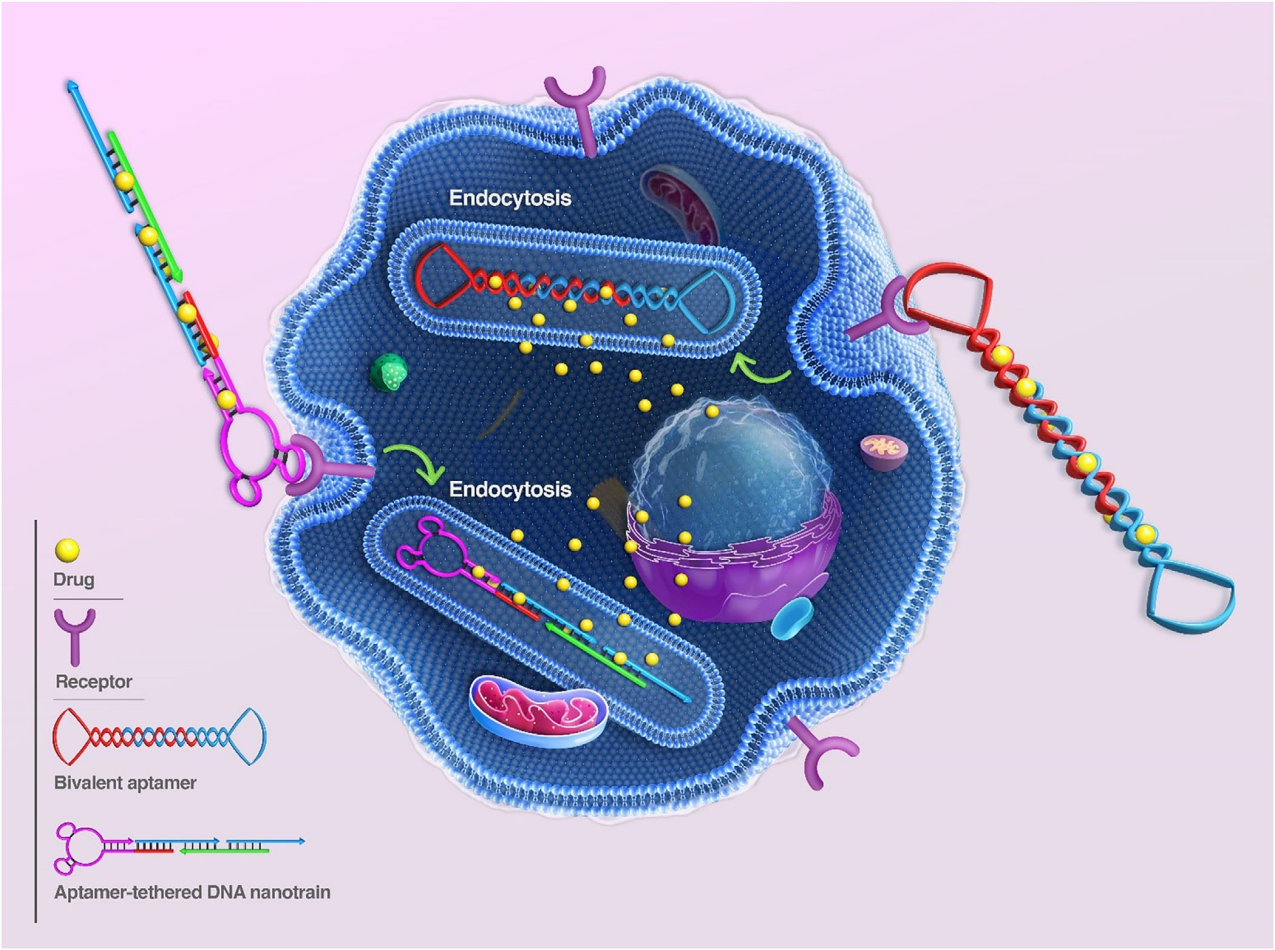
Schematic of drug delivery mechanisms based on DNA nanostructures integrated with aptamers that target cancer cells.

Inspired by aptamer‐based DNA nanostructures, researchers have presented diverse applications with promising prospects in various medical scientific fields such as drug delivery, sensing, diagnosis, scaffolding, and imaging. As the main content of this study, we highlight the different types of aptamer‐based DNA nanostructures, formation mechanism, application, and specific performance of these arrays in the various aspects of cancer diagnosis, drug delivery, imaging, and treatment, in vitro and in vivo. The study begins with a comprehensive discussion on the types and methods for forming aptamer‐based DNA nanostructures. After this, we provide a description and recent advances of nanostructures in cancer‐related therapeutic aspects. In the following, we mainly review and describe the application and performance of aptamer‐based DNA nanostructures in targeted drug delivery, gene therapy, and imaging. Finally, the current challenges and prospects of developing nanostructures for cancer diagnosis and treatment are presented to conclude this conducted study (Figure 1).

## 2 NANOSTRUCTURES

### 2.1 Nucleic acid nanostructures

The first DNA‐based structure was introduced in 1953 by Watson and Crick.^44^ To date, with the development and integration of biotechnology and nanotechnology knowledge, biomolecular engineers, inspired by the structural knowledge and propensity of nucleic acids (RNA/DNA) for self‐assembly or programmed assembly, in combination with aptamers, have been used to create a wide range of different nanostructures.^45^, ^46^, ^47^, ^48^ The self‐assembly approach is a process in which one or more molecules connect using non‐covalent interactions and form superstructures without the intervention of external factors.^49^ The predictable self‐assembly based on Watson‒Crick base pairs, high chemical stability, small diameter size, and helix spacing (about 2‒3.5 nm), makes DNA an ideal building block for the design and assembly of complex nanoscale structures with acceptable flexibility.^50^, ^51^, ^52^ In many studies, DNA nanostructures and aptamers have been introduced as designable structural components and selectable components, respectively.^53^, ^54^ Aptamers can be integrated with various DNA nanostructures through conjugation (covalent interactions) and hybridization (non‐covalent interactions).^55^, ^56^ The combination of these two components, while maintaining the useful properties of each infrastructure, has had a significant impact on the development of diagnostic and therapeutic platforms.^57^, ^58^

### 2.2 Mechanisms of aptamer‐tethered nanostructure formation

**FIGURE 2:**
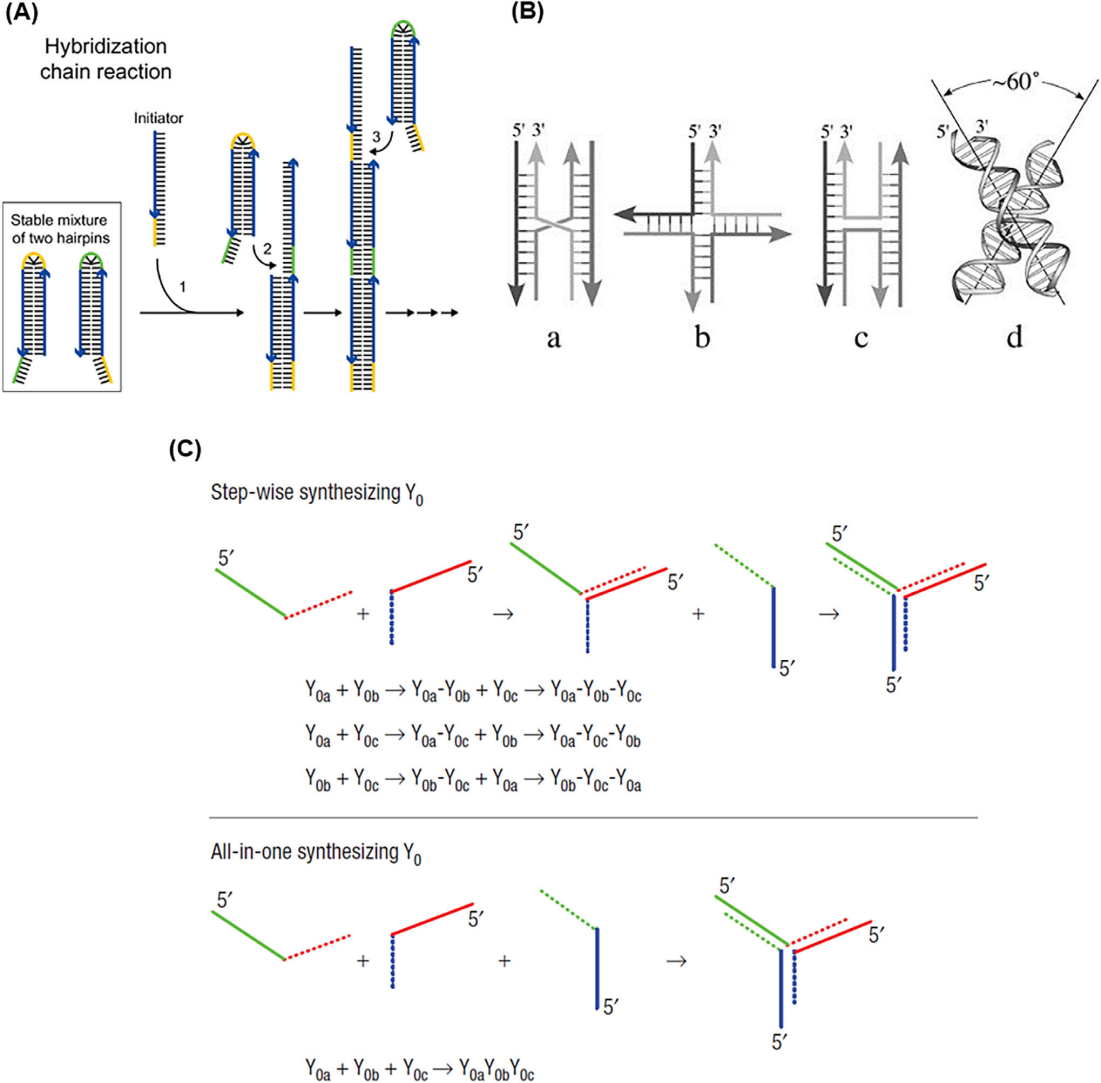
Summary of DNA nanostructures and assembly strategies. (A) Schematic of hybridization chain reaction (HCR) method. Adding an initiator strand with sequence‐specific to a stable mixture of two types of hairpins initiates the HCR event between the hairpins. Reprinted by permission from ref., 245 Copyright 2004 Springer Nature. (B) Overview of the DNA Holliday junction nanostructures. (a) The structure of the first DNA Holliday bond was introduced. (b) In the extended open form of this nanostructure, two crossed strands take a sharp U‐turn to connect the stacked duplex arms. (c) In the compact X‐stacked form of this nanostructure, four paired arms in two almost continuous helices are placed coaxially and are interrupted by the intersection of the strands. (d) In the model for the X‐stacked form, two pairs of DNA arms are in a positive rotation of about 60° to each other. Reprinted by permission from ref., 246 Copyright 2006 John Wiley & Sons, Ltd. (C) Schematic illustration of two three‐way junction (TWJ) assembly approaches: stepwise synthesis and one‐pot synthesis. The three arms of the DNA TWJ are named Y_0a_, Y_0b_, and Y_0c_. Reprinted by permission from ref., 100 Copyright 2004 Springer Nature.

One of the strategies usually used to assemble these nanostructures is based on the hybridization chain reaction (HCR) method (Figure 2A). HCR is an enzyme‐free isothermal amplification process initiated in the presence of an initiator (specific short ssDNA) and two trapped DNA hairpin structures. In this way, the initiator opens and activates the first hairpin. The sticky‐end of the first hairpin is exposed and hybridized with the second one through the formation of an interstrand base pair, which activates this hairpin. And so, the monomers are attached non‐covalently until the supply of hairpins is exhausted. This process results in long dsDNA polymers, formed by aggregation multiple monomers.^59^, ^60^

Rolling circle amplification (RCA) is an isothermal enzymatic nucleic acid amplification technology used to produce large‐scale DNA nanostructures. Building blocks consisting of very long single‐stranded strands with alternating sequences and pre‐designed structures and functions are the product of this amplification method.^61^ RCA products can be designed for various applications by varying the circular pattern sequence, leading to complex multifunctional DNA nanostructures, such as nanotubes,^62^ nanoflowers,^63^ and nanoribbons.^64^ Tile‐based assembly^65^ and catalytic hairpin assembly^66^ are other approaches used in this field. By organizing complementary strand pairs through Watson‒Crick base pairing, a myriad of diverse 1D, 2D, and 3D DNA nanostructures are generated in a predictable and tunable manner based on “bottom‒up” fabrication strategies.^67^, ^68^, ^69^, ^70^

The use of unique structural and functional properties of DNA nanostructures, such as tunable geometry, dynamic conformational switch, high drug loading capability, and high aspect ratio, has been welcomed in many diagnostic and therapeutic strategies.^71^, ^72^ Among the different forms of DNA nanostructures, DNA nanowires (NWs) are consequential designs for improvement of the analytical performance of the measurement system when the target concentration is very little.^73^ DNA NWs are usually made of two types of ssDNA with functional sites.^74^ Different from the RCA method that synthesizes DNA NWs through polymerase activity, the non‐enzymatic HCR method has been also used to assemble polymeric dsDNA NWs by cross‐activating DNA hairpin monomers.^75^, ^76^ Finally, the multivalent DNA NW can be simultaneously decorated with multiple DNA hairpins modified with diagnostic agents.^77^ Also, in the field of treatment and drug delivery, they have shown significant potential as an ideal theranostic agent.^78^ The basis of the concept of DNA nanostructures was began in 1982, when Seeman proposed the utilization of DNA structures to aid in the crystallization of proteins.^79^ Then, in 1998, a 2D DNA crystal structure was presented through the sticky‐end assembly of a two‐arm tile, revolutionizing the field of structural DNA nanotechnology.^80^ With extensive studies on the design of nucleic acid base pair sequences and specific hybridization of nucleic acids, 2D or 3D DNA nanostructures were designed.^81^, ^82^ During various amplification steps, such as the RCA method for synthesis of DNA nanostructures, if the sequences are overstretched, they will no longer be able to maintain their linear structure. Therefore, they become bent or intertwined such as DNA nanoclaw, which can be classified as “other” DNA nanostructures compared to other organized DNA nanostructures.^83^ Various functional aptamers can be conjugated to DNA nanoclaw structure,^84^ which provides multivalent spatial patterns of aptamers, suitable for fabricating sensors with improved detection performance (achieving simultaneous detection), selectivity, and sensitivity by reducing signal interference.^85^, ^86^ For example, Wang et al.^87^ presented an attractive platform that used magnetic rigid flexible DNA nanoclaws inspired by octopus arms to improve cancer cell uptake (up to 82.3 ± 7.1%). Also, a three‐arm nanoclaw aptamer assembled for accurate diagnosis and improved tumor therapy was presented.^88^ With the development of geometric shapes, the formation of multi‐helical junctions (loops) was achieved from the convergence of three, four, or more complementary nucleic acid strands.^89^ Due to its structural stability and flexibility, it has become an attractive and widely used building block for the construction of diagnostic platforms.^90^

In 1964, the conformation and structure of the DNA Holliday junction were first introduced by Holliday.^91^ Then, in 1982, Seeman presented a Holliday‐shaped cross‐linking structure that results from joining four synthetic arms of DNA through sequence symmetry breaking.^79^ These nanostructures occur temporarily during the process of DNA recombination (meiosis and mitosis).^92^ A year later, with Holliday binding established, they introduced synthetic immobilized DNA four‐way junctions (DNA FWJs), which consist of two parallel DNA double helices, without deleterious double symmetry, bounded by two cross‐links.^93^ In 1993, Seeman et al.^94^ put two double helix structures together to be bound by only one crossover. This structure is made of four ssDNA with a unique sequence, which prevents strand migration and gives acceptable stability to the structure by minimizing the symmetry of the sequence at the junction. According to the type and concentration of cations in the solution, DNA FWJ is created in two main forms: open conformation, which occurs at low salt concentrations, and a square‐planar shape that forms in the middle of the binding site. In another form, a more compact X‐stacked conformation occurs at high salt concentrations where two pairs of DNA arms are stacked coaxially (with an angle of about 60° to each other) (Figure 2B).^90^

Meanwhile, DNA three‐way junctions (DNA TWJs) have a simpler topological nanostructure with flexibility and a controllable shape.^95^, ^96^, ^97^ DNA TWJs are completely base paired, the three angles between their arms are approximately equal, and they fold into a well‐defined conformation in the presence of divalent ions, forming a flexible and even rigid pyramidal structure.^98^, ^99^ In 2004, Li et al.^100^ introduced two approaches for assembling a Y‐shaped DNA nanostructure (Figure 2C). In stepwise synthesis, two DNA strands with complementary sequences at each end are hybridized that form an arm of a DNA TWJ structure. Then, another DNA strand is added and hybridized with the two unconnected ends of the previous strands, which completes the DNA TWJ structure. An all‐in‐one synthesis (one‐pot) is simpler than the previous method, and three strands of DNA with complementary sequences at each end are mixed in an equal molar ratio, which forms the DNA TWJ structure. It should be noted that no difference in the structure and functionality of the products of these two approaches has been observed. Since no significant degradation was observed at 4°C after 30 days, the synthesized DNA TWJs possess high stability. In combination with aptamers, it has appeared very successful in various fields, such as targeted delivery of therapeutic agents and increasing the sensitivity of diagnostic platforms by reducing the incubation time.^101^, ^102^ For example, Wu et al.^103^ designed a multifunctional aptamer‐based DNA nanoassembly using the modification of the functional arms of DNA TWJs. These arms were programmed with aptamer, intercalated anticancer drugs, and antisense oligonucleotides. This complex provides a high potential for the diagnosis and targeted treatment of cancer along with a 1000‐fold loading of anticancer drugs and imaging agents. Also, by engineering functional arms, it could be simultaneously integrated fluorescent, colorimetric, and electrochemical properties.^104^

**FIGURE 3:**
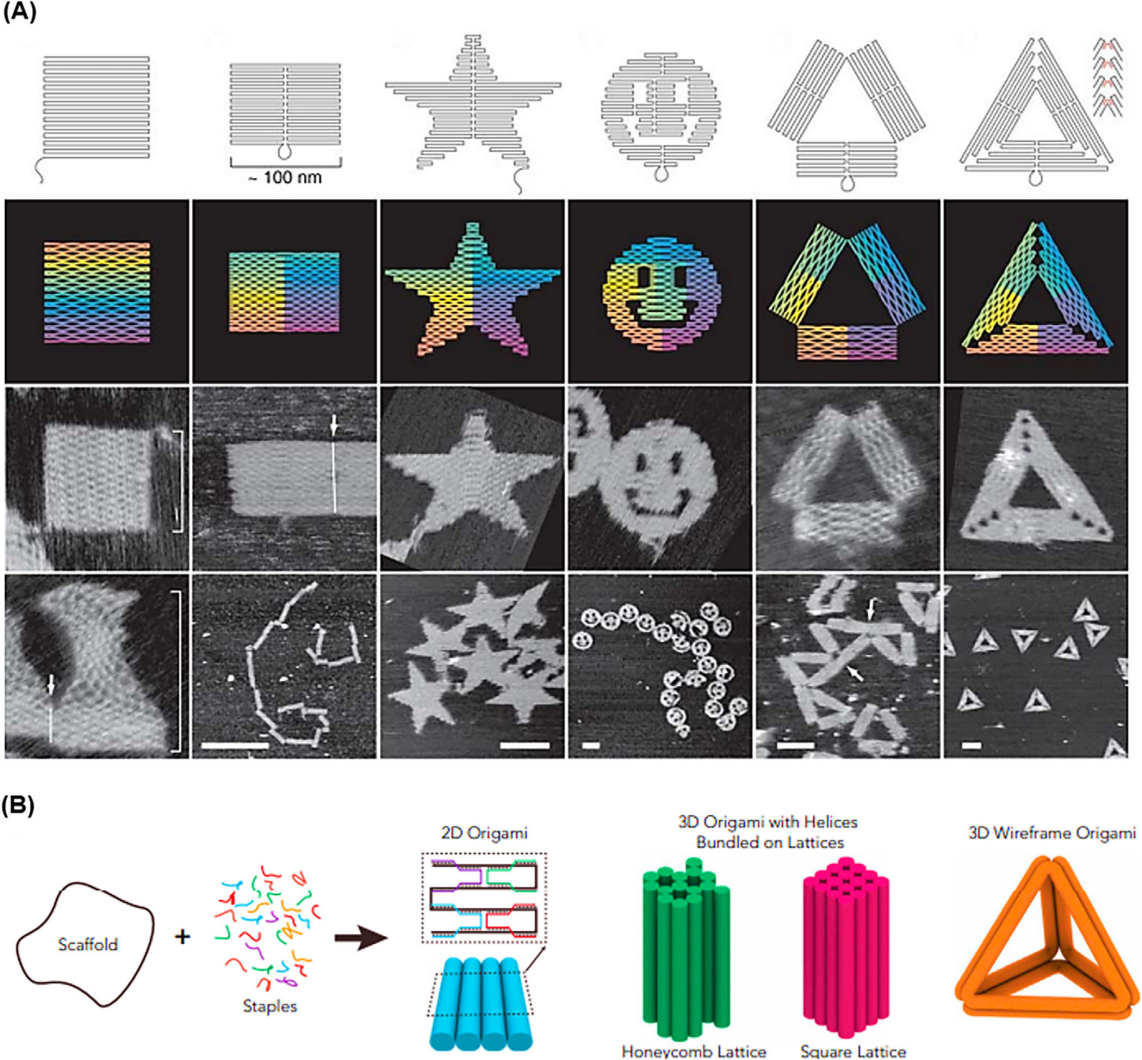
Schematic of the assembly hierarchy of different 2D and 3D DNA origami nanostructures. (A) Long single‐stranded DNA (ssDNA) strands are designed to connect distant segments through cross‐base pairing to complement different parts of the scaffold DNA. Reprinted by permission from ref., 105 Copyright 2006 Springer Nature. (B) Different shapes of 2D DNA origami nanostructures, in order from left to right: square, rectangle, star, disc with three holes, triangle with rectangular domains, and acute triangle with trapezoidal domains. Reprinted by permission from ref., 247 Copyright 2017 Elsevier.

In 2006, Rothemund^105^ used the inherent self‐assembly property of DNA molecules to create highly complex nanostructures called DNA origami. To construct the proposed DNA origami, a long ssDNA scaffold and more than 200 short main strands complementary to the linear distal sequences of the scaffold strand were used to fold the strand into larger and more complex desired shapes that self‐assemble in a single step (Figure 3A). Usually, for the long ssDNA scaffold, M13 phage genomic DNA is used, which is about 7000 bp, and the short main strand is about 20‒60 bp long.^106^ The output of this process is programmable and organized scaffolds such as triangles,^107^ discs,^108^ five‐pointed stars,^109^ and smile symbols.^110^ These shapes usually have a diameter of about 100 nm with a spatial resolution of 6 nm.^105^ In 2009, Douglas et al.^111^ proposed 3D DNA origami structures by folding 2D flat DNA origami sheets. Three‐dimensional DNA origami assembly was performed through DNA helices in a honeycomb network by one‐pot synthesis. A mixture of a single strand of scaffold and hundreds of main strands of DNA is rapidly heated and then allowed to cool slowly. This process directs the folding of the strands toward the desired main structure (Figure 3B). To increase the efficiency of the DNA origami machine, aptamers have been successfully integrated into this structure to build a smart therapeutic, diagnostic, and imaging system.^112^, ^113^ For example, Chen et al.^114^ integrated different types of aptamers (such as AS1411, MUC1, and EpCAM) on the surface of triangular DNA origami structures to detect cancer cells. This sensing strategy was able to identify discriminately blinded unknown cancer cells with high sensitivity and detection accuracy (95.0%).

**FIGURE 4:**
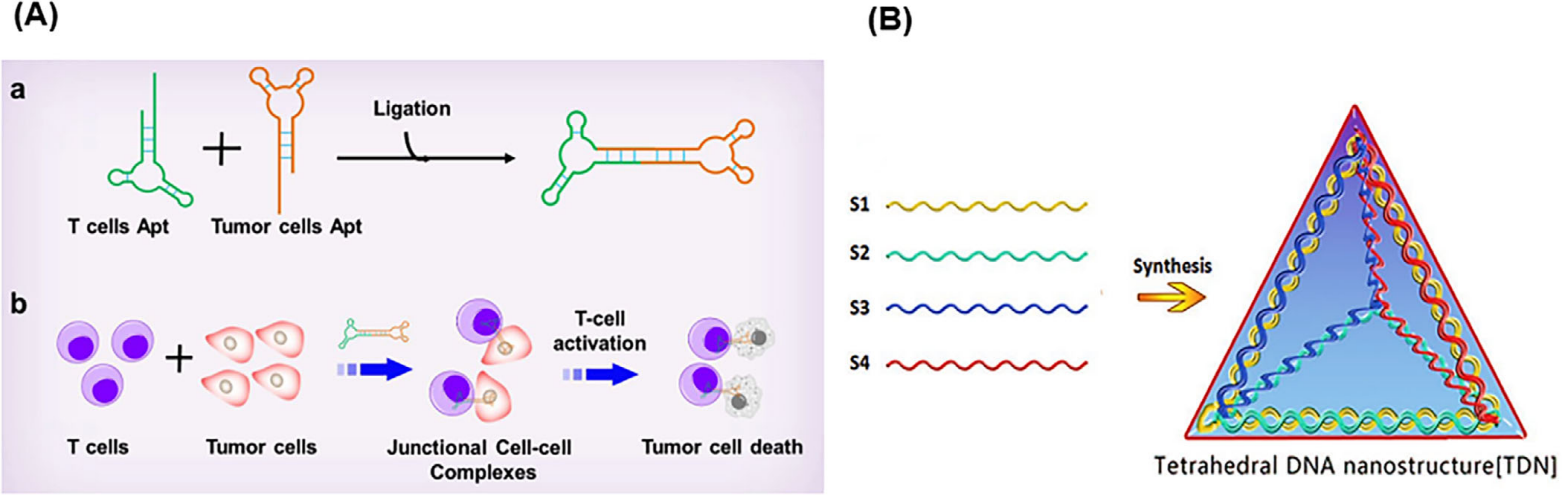
Nanostructures for biomedical applications. (A) Overview of circular bivalent aptamer (Cb‐Apt) construction steps and function for targeted cancer immunotherapy. (a) Construction of Cb‐Apt hybridization of two different types of aptamer sequences through T4 DNA ligase. (b) The function of Cb‐Apt is to simultaneously recognize T cells and cancer cells and mediate the formation of junctional cell‒cell complexes to kill cancer cells. Reprinted by permission from ref., 118 Copyright 2020 American Chemical Society. (B) Schematic representation of DNA tetrahedral. The four single‐stranded DNAs (ssDNAs) used in the structure are named S1, S2, S3, and S4. Reprinted by permission from ref., 135 Copyright 2017 John Wiley & Sons, Ltd.

A DNA nanostructure with a cyclization method has been reported for the stabilization of aptamers by ligation of the two ends of aptamer‐based oligonucleotides, achieving circular bivalent aptamers (Cb‐Apts). It significantly improves the recognition ability and molecular binding affinity compared to monovalent aptamers because identifying one of the ligands can increase the local concentration and binding of the remaining ligands.^115^, ^116^, ^117^ One of the most important features of the Cb‐Apt systems is their resistance to nucleases due to the lack of free 3ʹ‐ and 5ʹ‐ends, which improves their thermal stability, physical stability, blood circulation time, and half‐life.^118^, ^119^ Several environmental factors, such as metal ions concentration, temperature, pH, and nuclease enzyme, can have a significant effect on such aptamer‐integrated DNA nanostructures.^120^, ^121^ Kuai et al.^117^ reported that annealing conditions significantly affect the formation of a Cb‐Apt such that quick chilling of denatured aptamer, compared to slow chilling, effectively facilitates the formation of a Cb‐Apt. Also, aptamers with additional 13 bp complementary sequences at the 5ʹ‐end have better binding ability than the same aptamer with additional 9 or 17 bp complementary sequences. Moreover, systematic studies have shown that Cb‐Apt has nucleation resistance, thermal stability, and increased affinity in biological environments, also improving the aptamer efficiency for cancer diagnosis and in vivo treatment.^122^ In another study, Yang et al.^118^ presented Cb‐Apt to improve cancer immunotherapy. In this strategy, two different types of aptamers were designed for simultaneous targeting of T cells and cancer cells (Figure 4A). In this approach, Cb‐Apt as a mediator led to the facilitation of T‐cell recognition function to target tumor cells. To prove the concept, LD201t1 aptamer (specific for CD62L, expressed on the surface of the T‐cell membrane) and sgc8 aptamer (specific for PTK7, expressed on the surface of cancer cells) were used in the presented Cb‐Apt. Both types of the aptamers were designed in such a way that they lacked free ends by ligating 3′‐ and 5′‐ends and a 13 bp additional flanking complementary sequence and were conjugated through T4 DNA ligase. Multivalent aptamers are multifunctional molecules constructed from two or more identical or different aptamer motifs that may or may not have structural elements or functional groups. This strategy is ideal for building high‐sensitivity platforms for the simultaneous treatment or detection of multiple analytes.^123^, ^124^

Another attractive drug transport platform is based on aptNTrs nanostructures as long dsDNA structures that self‐assemble via the HCR method.^125^ The aptNTrs are novel as one of the most promising nanoconstructs for the efficient targeted delivery of therapeutic agents while minimizing side effects. This is due to their ease of synthesis, re‐programmability, enhanced maximum tolerated dose (MTD), biodegradability, high drug loading capacity, and ability to control drug release.^126^, ^127^ An aptNTr is simply constructed from two partially hairpin DNA building blocks. In the absence of the initiator probe, the energy stored in each ring of monomers, M1 and M2, is retained by the corresponding stem, which prevents their hybridization and polymerization. Primer DNA probes bind to the aptamer moiety at one end of the nanostructure and induce cascade hybridization between M1 and M2 via the HCR method.^128^ The product of this process is a highly ordered and long duplex DNA with numerous addressable sites where various therapeutic and diagnostic agents can be located or anchored on these helices, allowing high‐capacity loading of therapeutic agents.^129^

DNA cube was the first polyhedron synthesized in 1991 by a multistep process,^130^ followed in 2004, tetrahedral DNA nanostructure (TDN) was synthesized using four short oligonucleotides of equal molar ratio, via a single‐step method; and a revolution in therapeutic applications of polyhedron nanostructures appeared.^131^, ^132^ TDNs have strong mechanical strength, high precision, and ease of preparation, and have shown wide prospects in practical applications such as ideal infrastructure for biosensors and screening and early diagnosis of clinical diseases.^133^, ^134^ To make TDN, four ssDNAs with specific sequences are used in equimolar amounts (Figure 4B). Each ssDNA forms a triangle, and three sides of this triangle hybridize with the complementary sequence located on the other three ssDNAs. Each side of the triangle hybridizes with the side of the other triangle, finally forming a 3D TDN with a double helix structure.^135^ Usually, the TDN is formed by a simple and automatic method through a one‐step annealing operation. In this way, four ssDNAs are incubated in a buffer solution consisting of Tris and MgCl_2_ at 95°C for 10 min and then, cooled at 4°C for 30 min.^136^, ^137^ With the intelligent engineering of this structure, Wang et al.^138^ introduced a DNA logic‐gated nanorobot to identify different cells based on cell surface biomarkers. TDNs showed acceptable stability in the biological environment (for 42 h).^139^

In addition to the TDNs, DNA polyhedral nanostructures, such as DNA cubes, dodecahedrons, and buckyballs, were created using symmetric three‐point‐star motifs. The DNA octahedron and icosahedron were generated using symmetric four‐point‐star motifs and five‐point‐star motifs, respectively.^140^, ^141^ For example, the octahedral nanostructure of DNA was constructed by folding a ssDNA about 1600 bp long by complementary pairing in the presence of five 40 bp synthetic ssDNAs.^142^ However, one of the weaknesses of these forms is selectivity for binding and targeted drug delivery. To solve this problem, aptamers were integrated with the DNA octahedral nanostructure; for example, specific aptamers were conjugated to the desired strands using a spacer linker.^143^, ^144^, ^145^ Among other representatives of DNA nanostructures with high functional sites, there are DNA nanosponges,^146^ nanoclews,^147^ and nanocapsules.^148^ In these forms, long RCA strands are assembled into different forms by liquid crystallization and corresponding DNA building blocks. Usually, DNA nanosponges integrated with aptamers are obtained through one‐pot synthesis using the RCA method.^149^ During the assembly process of RCA‐based nanostructures, functional elements such as fluorescent labels can be inserted into this structure.^150^ Also, these structures, as non‐nicked building blocks, have shown acceptable stability in physiological and biological environments.^150^, ^151^ This dense nanostructure ensures a large loading capacity of therapeutic agents with a safe and effective delivery approach and high biological stability.^152^ Inspired by this strategy, Zhang et al.^146^ designed a self‐assembled DNA nanosponge based on RCA with gene silencing and antitumor effects. This compact structure effectively and simultaneously delivered many antisense oligonucleotides and doxorubicin (DOX) for intracellular uptake and clearance of miRNA‐21 with high efficiency.

## 3 CLASSIFICATION BASED ON BIOMEDICAL APPLICATIONS

Aptamer‐tethered nanostructures have appeared promising in various aspects of therapy and have overcome the limitations of conventional anticancer therapies. Here, a detailed study is presented on the current status of aptamer‐tethered nanostructures used in various treatment strategies such as drug delivery, oligonucleotide therapy, and bioimaging. Relying on the logic, principles, and advantages of aptamer‐tethered nanostructures in combination therapy, chemodynamic therapy (CDT), and cytotoxic proteins, we can better understand the development of targeted nanoplatforms to improve treatment. Also, the effect on the cell nucleus and disruption of gene expression are investigated as targeting parts for delivery of oligonucleotide drugs through aptamer‐tethered nanostructures. On the other hand, these nanostructures in bioimaging using chlorin e6 (Ce6) and anthracyclines, molecular beacons (MBEs), and single‐photon emission computed tomography (SPECT) improve the insight of the treatment process by monitoring the effect of therapeutic agents and understanding their function.

### 3.1 Drug delivery

Although significant progress has been made in the field of cancer diagnosis and treatment, these methods have still not been effective in treating this malignancy.^153^ Chemotherapy is one of the main methods for cancer treatment, but in addition to toxicity on cancer cells, it has serious and unpredictable side effects on healthy cells.^154^, ^155^ Some adverse side effects, such as digestive disorders, hair loss, drug resistance, and cardiac toxicity, unfortunately, can lead to a reduction in dosage or even discontinuation of treatment.^156^, ^157^ Traditional chemotherapy methods suffer from several limitations, such as poor biodistribution and non‐specific targeting. Besides, they usually cannot distinguish healthy cells from tumor cells, reducing the effectiveness of treatment. Basically, the identification of target cell surface receptors with high precision requires the development of targeted drug delivery platforms.^158^, ^159^ All kinds of oral pills and common simple injections are not able to meet the needs of targeted and practical treatment because about 90% of the prescribed drugs affect healthy cells and only 2%‒5% are internalized by cancer cells.^157^, ^160^ In addition, the insufficient therapeutic outcome of anticancer medications arises from their inherent characteristics, such as limited solubility in water, instability, and quick metabolism accompanied by an irritating property. Henceforth, to enhance the effectiveness of these drugs, carriers are required to safeguard and deliver them precisely to the tumor location.^161^, ^162^ The carriers used for drug delivery should be essentially non‐toxic and possess the ability to be modified for high drug‐loading capacity. Additionally, they should have a long circulating half‐life and undergo simple and cost‐effective manufacturing steps. These properties lead to a reduction in drug dosage by enabling the delivery of effective drug concentrations specifically to the tumor site. As a result, they contribute to minimizing side effects and clinical costs while improving the treatment process and the patient's quality of life.^163^, ^164^ Various types of drug carriers have been investigated for targeted drug delivery, including liposomes,^165^ micelles,^166^ and carbon nanotubes.^167^ Additionally, various antibodies, peptides, and proteins have been explored as ligands for targeting specific receptors on cancer cells. However, there are many disadvantages, such as low stability, high cost, complex manufacturing steps, low drug loading, immunogenicity, low binding affinity, and high drug release rate, causing the ineffectiveness of these methods.^168^, ^169^, ^170^, ^171^, ^172^ Therefore, the development of a safe drug carrier is necessary for targeted drug delivery and localized tumor treatment, aiming to reduce side effects. In this regard, DNA nanostructure have been investigated as safe and compatible vehicles for targeted drug delivery to solid tumors.^173^, ^174^ Considering the therapeutic goals and challenges related to the treatment, various aspects of targeted drug delivery, such as chemotherapy, combination therapy, CDT, and cytotoxic proteins have been successfully designed and applied to increase anticancer activity. According to the potentials mentioned, DNA nanostructures can become one of the primary targets for cancer treatment in the near future. In the following, we will discuss some recent developments in this field.

#### 3.1.1 Chemotherapy

**FIGURE 5:**
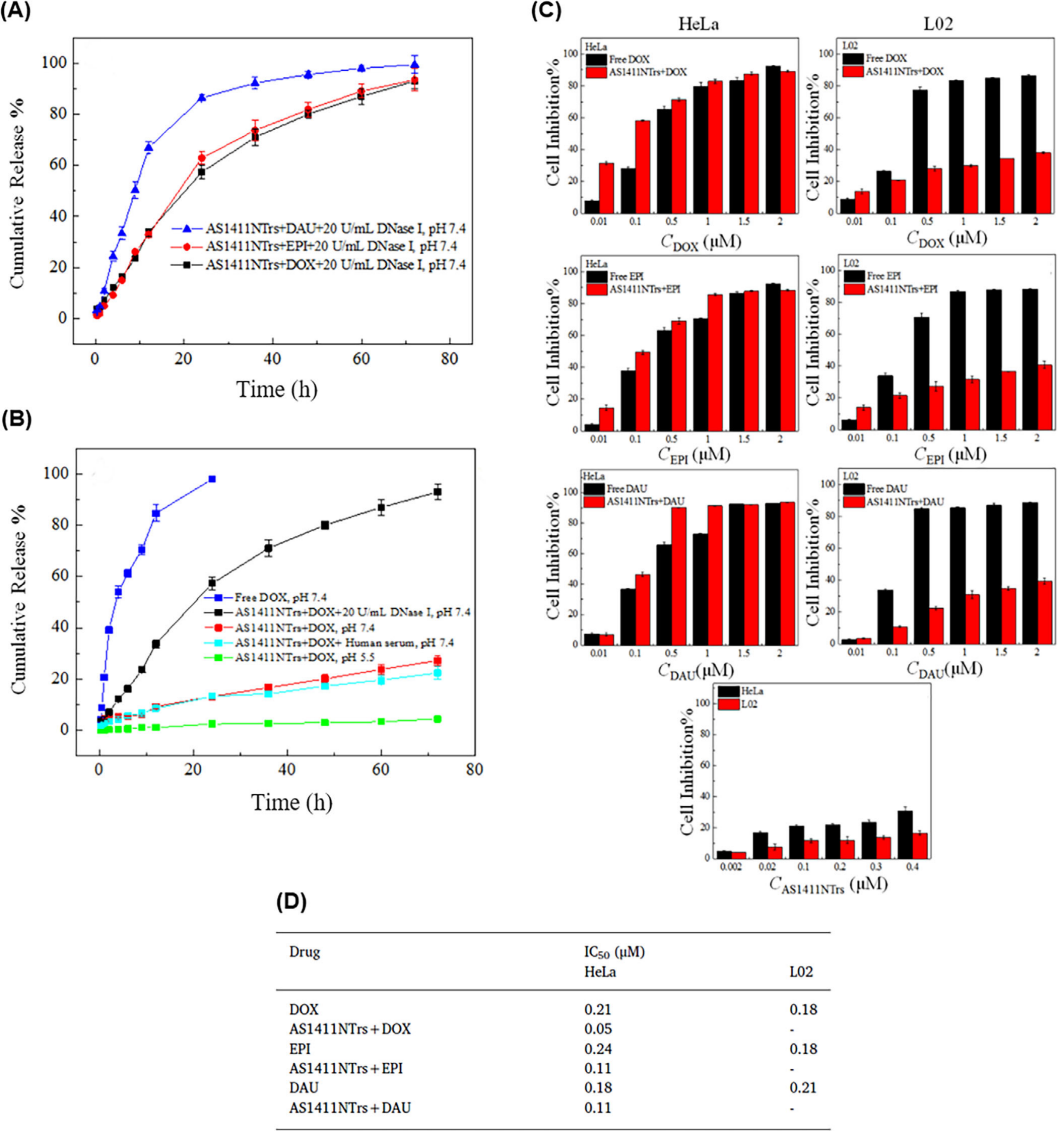
Drug release and cytotoxicity profiles of AS1411NTr for HeLa cells. (A) The release rates of three drugs, doxorubicin (DOX), epirubicin (EPI), and DAU, in the medium containing DNase I (20 units/mL) up to 72 h. (B) Release profile of DOX with a concentration of 30 µM (in vitro conditions) and in complex with AS1411NTrs at different conditions. (C) Comparison of cytotoxicity (AS1411NTr containing DOX and free DOX, AS1411NTr containing EPI and free EPI, and AS1411NTr containing DAU and free DAU) on the target cells (HeLa, nucleolin positive) and non‐target cells (L02, nucleolin negative). (D) Calculated IC_50_ values for DOX, EPI, and DAU alone and DOX, EPI, and DAU together with AS1411NTr in HeLa cells and L02 cells. Reprinted by permission from ref., 176 Copyright 2020 Elsevier.

From the perspective of drug delivery, overcoming the challenge of degradation by nucleases while achieving effective drug delivery with specific targeting of tumor cells has always been a priority. Hence, Xue et al.^175^ constructed a DNA NW with a core‒shell nanostructure composed of two fundamental structural units, each assembled from six ssDNAs and arranged in a head‐to‐tail manner, named DNA NWs‒aptamer. The dsDNA helices served as the core, while the standing hairpin DNA aptamers functioned as the shell, protecting the entire nanostructure from nuclease degradation. The advantage of this design is that all terminals are located in the core area, hidden from nuclease attacks, while the specific binding sites for target molecules are exposed in the shell area, making this nanostructure resistant with high selective targeting capability. Stability assessments conducted in fetal bovine serum showed that 80% of DNA NWs‒aptamer remained intact after 24 h of incubation. In vivo distribution also demonstrated an extended circulation time in the bloodstream for DNA NWs‒aptamer. Each unit of DNA NWs‒aptamer carries 64 DOX molecules, indicating a high drug‐loading capacity. Cell viability assessments showed that NWs‒aptamer‒DOX resulted in less than 40% viability in CEM cells, while it maintained 90% viability in Ramos cells. Additionally, it induced 22.2% cell apoptosis in CEM cells, whereas 97% of these cells remained viable when exposed to NWs‒aptamer formulations without DOX. In vivo therapeutic efficacy demonstrated 70% tumor suppression in human cervical cancer (HeLa) cells treated with NWs‒aptamer‒DOX, highlighting the potential of this nanostructure for cancer chemotherapy. To reduce the systemic toxicity of chemotherapy drugs, Pei et al.^176^ designed aptNTrs (AS1411NTr) for HeLa cells. They composed it of a modified aptamer with a probe (AS1411‐P1) and hybridized it with two hairpin monomers (as a box car), H1 and H2, through an HCR method. Among these tested anthracycline drugs, DOX had the highest binding constant, while daunorubicin (DAU) possessed the highest drug release rate constant at pH 7.4 (DAU > EPI > DOX). It can be concluded that there was an opposite relationship between the binding affinity and drug release in the nanotrain. In the medium containing endonuclease (DNase I), the maximum release for all drugs was ∼100%, showing that the endonuclease present in the cell lysosome could cause the drug release (Figure 5A). Also, after 72 h, the release of DOX from the nanocomplex in human serum was about 22.3%, which confirmed the stability of this system (Figure 5B). Nanotrain loaded with the drugs showed very little cytotoxicity in non‐target cells (normal human liver L02 cells) compared to the target cells (HeLa) (Figure 5C). Also, the IC_50_ values were calculated for DOX, epirubicin (EPI), and DAU along with AS1411NTrs, in HeLa cells, as 0.05, 0.11, and 0.11 µM, respectively. They were less than the IC_50_ values of free DOX (Figure 5D). The nanotrain will have bright prospects in the future due to its high ability to load different drugs, specific binding and targeted drug delivery, and low drug toxicity on healthy cells.

**FIGURE 6:**
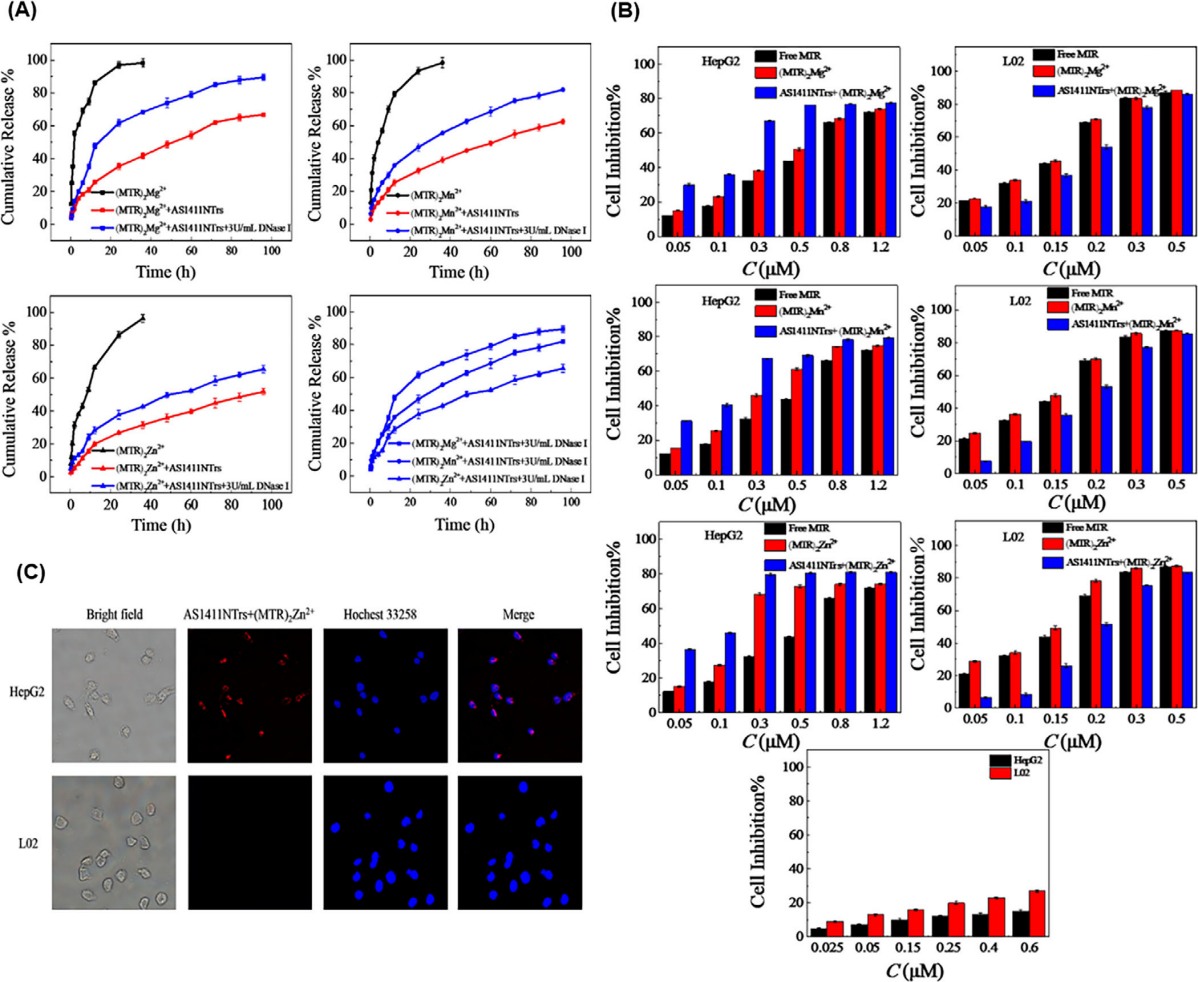
Evaluation of metal ion dimers loaded on AS1411NTr for targeted delivery. (A) Drug release rate of (MTR)_2_Mg^2+^, (MTR)_2_Mn^2+^, and (MTR)_2_Zn^2+^ dimers from AS1411NTr in the absence and presence of DNase I. (B) Cytotoxicity of free mithramycin (MTR), dimers, and dimer complex loaded onto AS1411NTr in HepG2 (nucleolin positive) and L02 (nucleolin negative) cells. (C) Fluorescence microscopic images of the accumulation of AS1411NTr loaded by (MTR)_2_Zn^2+^ in the nucleus of HepG2 cells compared to L02. Reprinted by permission from ref., 177 Copyright 2021 Elsevier.

As mentioned, the binding affinity of a drug to a nanotrain has an essential effect on its loading, release, and effectiveness. Wang et al.^177^ used divalent ions to enhance the binding of a drug to the nanotrain. Divalent ions such as Mg^2+^, Zn^2+^, or Mn^2+^ can interact with the oxygen atoms in the chromophore of two mithramycin (MTR) molecules, forming dimer complexes ((MTR)_2_Mg^2+^, (MTR)_2_Zn^2+^, and (MTR)_2_Mn^2+^). The inclusion of divalent ions improved selectivity and facilitated the binding of MTR to the CG‐rich regions of the nanotrain that was attached to the AS1411 aptamer. Similar to previous methods, AS1411NTr consisted of the H1 and H2 sequences, which were hybridized to the AS1411 trigger (AS1411‐P1) through an HCR reaction. The release rate of the dimer loaded on the AS1411NTr was slower than that of free MTR dimers. The release rate increased with the addition of DNase I, which may be due to DNA hydrolysis by endonucleases (Figure 6A). IC_50_ values based on MTT assay for AS1411NTr containing (MTR)_2_Mg^2+^, (MTR)_2_Mn^2+^, and (MTR)_2_Zn^2+^ in human liver cancer cells (HepG2, nucleolin positive) were 0.16, 0.15, and 0.10 µM, respectively. While this value for human normal hepatocyte cells (L02, nucleolin negative) was 0.17, 0.18, and 0.22 µM, respectively. According to the reported results, AS1411NTr containing MTR dimers has strong cytotoxicity and robust targeting ability for HepG2 cells, especially for (MTR)_2_Zn^2+^ (Figure 6B). Among the three reported metals, (MTR)_2_Zn^2+^ exhibited a higher affinity for AS1411. Moreover, AS1411NTr with proper targeting was able to powerfully inhibit cancer cells while reducing the toxic effects on healthy cells (Figure 6C).

**FIGURE 7:**
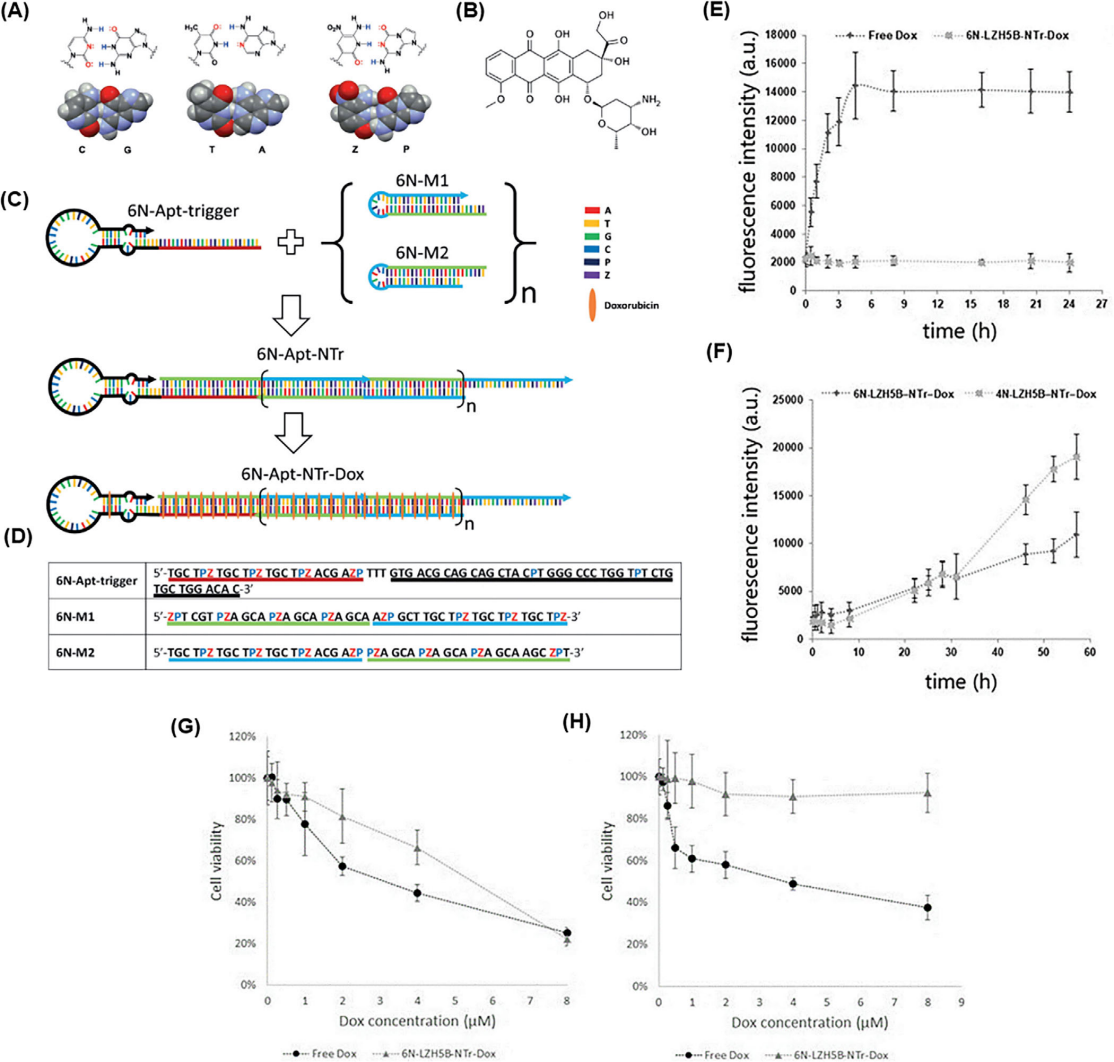
Components and construction steps of 6N‐LZH5B‐NTr‐DOX. (A) Chemical structure of A:T, C:G, and P:Z base pairs. (B) Chemical structure of the doxorubicin (DOX) drug. (C) The probe sequence (in red) was ligated to the 5ʹ‐end of the 6N‐Apt‐trigger aptamer sequence (in black) and then, hybridized to the 6N‐M1 and 6N‐M2 hairpins (in blue). In this way, it first was hybridized to the 6N‐M1 base and then, attacked its hairpin and produced a strand, which subsequently attacked the 6N‐M2 hairpin. DOX (in orange) was loaded in these areas. (D) The sequence of nucleotides in the six‐letter DNA. (E) Fluorescence emission of DOX carried by six‐letter DNA and free DOX, and (F) DOX carried four‐letter DNA. (G) The cell viability assay of free DOX and 6N‐LZH5B‐NTr‐DOX in the target cells (HepG2), and (H) non‐target cells (Hu1545). Reprinted by permission from ref., 178 Copyright 2020 John Wiley & Sons, Ltd.

In another study, Zhang et al.^178^ developed an aptNTr engineered with genetic alphabet changes specifically for liver cancer (HepG2) cells. By incorporating an artificially expanded genetic alphabet, they introduced two additional nucleotides (P:Z pairs) to the existing set of sequence nucleotides, mimicking the shape and size of natural nucleotide pairs (G:C and A:T) (Figure 7A). Increasing the number of nucleotides in DNA led to an enhancement in the density of functional DNA information and protection against the attack of natural DNA/RNAs in the system. To construct the six‐letter DNA (GACTZP) as a carrier for the DOX drug (Figure 7B), the trigger probe was initially attached to the 5ʹ‐end of the modified aptamer (LZH5B). Then, two hairpin monomers (6NM1 and 6NM2) were introduced to the end through trigger‐induced mutual hybridization (Figure 7C,D). The resulting nanotrain (6N‐LZH5B‐NTr‐DOX) demonstrated greater stability compared to the standard four‐letter DNA. Furthermore, the release rate of DOX loaded on 6N‐LZH5B‐NTr significantly decreased in comparison with DOX alone (Figure 7E,F). In target cells, after the effect of 6N‐LZH5B‐NTr‐DOX and free DOX, the viability of cells in both groups was about 20%. But it was about 90% and 40% in non‐target cells, respectively. This indicates equal cytotoxicity of these two groups on cancer cells and much less adverse effects of 6N‐LZH5B‐NTr‐DOX than free DOX on normal cells (Figure 7G,H). The proposed nanotrain with high stability, selective cell recognition, and non‐targeted toxicity will have promising prospects in the future.

**FIGURE 8:**
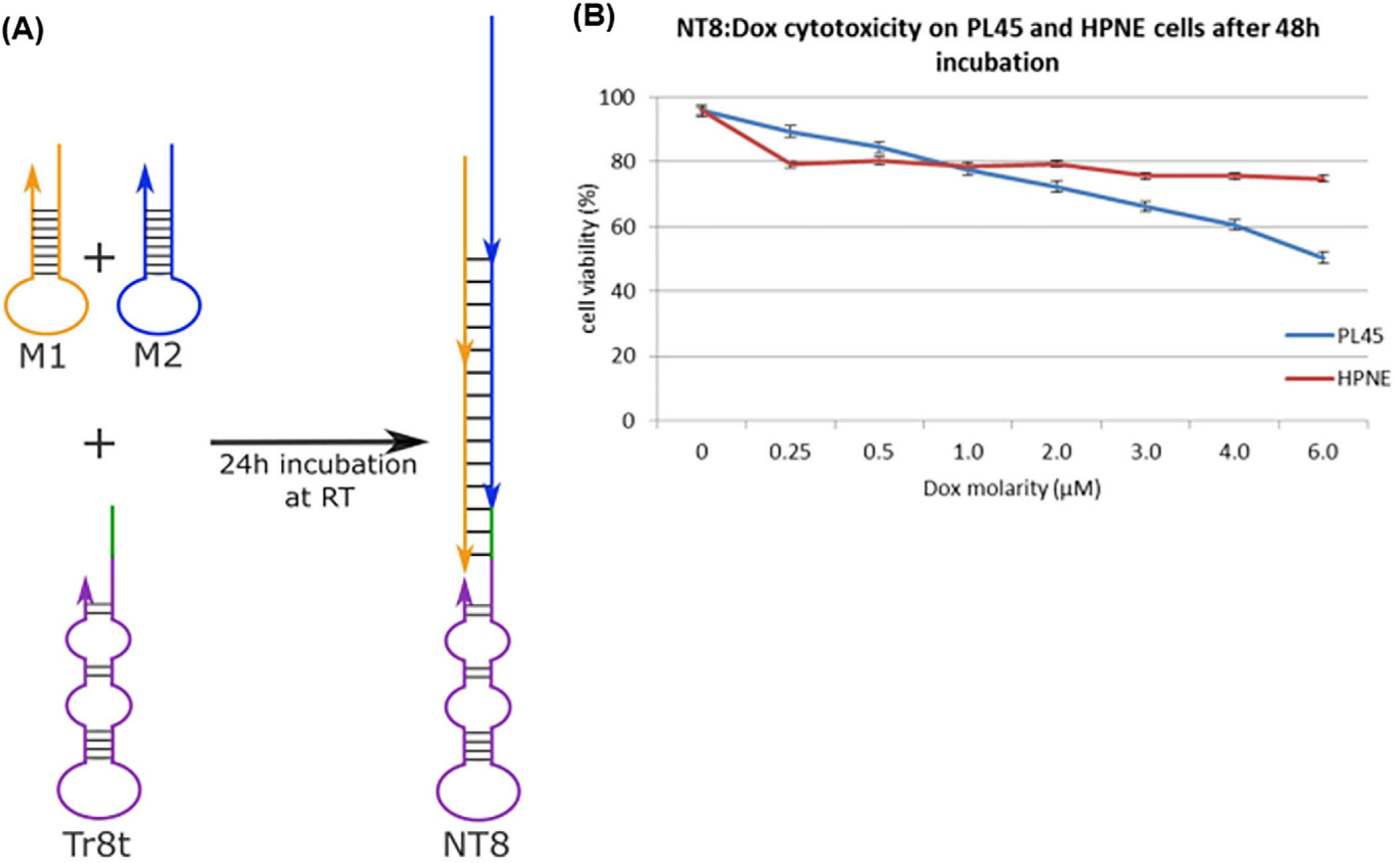
Design and therapeutic effects of the NT8 nanotrain. (A) Nanotrain design, the single‐stranded sequences of M1 and M2 along with aptamer modified with the trigger (Tr8t) were incubated for 24 h and NT8 nanotrain was obtained. (B) Effects of NT8 loaded with doxorubicin (DOX) on PL45 and (HPNE) were measured by the MTS assay. Reprinted by permission from ref., 179 Copyright 2015 Springer Nature.

The preservation of the targeting property of aptamer‐modified nanocarriers and their significant cytotoxicity with minimal side effects can be considered an effective approach for the early detection and treatment of cancer. Hence, Champanhac et al.^179^ reported a PL8 aptNTrs combined with the chemotherapeutic DOX drug, for the treatment of human pancreatic ductal adenocarcinoma (PL45). The specific trigger sequence of the PL8 aptamer, which was designed to target PL45 cells, induced a reaction in the HCR system. This led to the hybridization of the aptamer with both M1 and M2 sequences (DOX loading site). As a result, a nanotrain (NT8) formed, as depicted in Figure 8A. Changes in intracellular pH and the presence of nucleases led to the controlled release of DOX from the drug delivery system. Cell viability of both target cells (PL45) and healthy cells (HPNE) was evaluated 48 h after treatment with NT8 loaded with DOX. The viability of target cells was measured to be 50%, while the viability of healthy cells was found to be above 75% (Figure 8B).

Bladder cancer treatment with chemotherapy drugs often leads to intravesical recurrences and side effects. Wang et al.^180^ introduced a nanotrain (B1 NT) for the first time to overcome these problems, incorporating the bladder cancer‐specific aptamer (B1). The nanotrain structure is formed by binding the aptamer B1 to the C/G nucleotide‐rich sequences, known as M1 and M2. These sequences contain sites for loading EPI. EPI belongs to the group of anthracyclines with intrinsic fluorescence properties. It is widely recognized as one of the most effective therapeutic drugs for intravesical bladder cancer. After being placed in two DNA strands, its fluorescence is turned off. This feature was used to investigate drug loading and drug release in laboratory conditions. B1 NT‐EPI entered the T24 bladder cancer cells through endocytosis and micropinocytosis, facilitated by the clathrin‐mediated pathway. The high targeting capability and selective toxicity of B1 NT‐EPI were confirmed by fluorescence signal analysis and confocal images of the bladder cancer cells (T24 and KU‐7) and normal bladder urothelial cells (SV‐huc‐1) after their treatment with B1 NT‐EPI. Furthermore, it was noted during in vitro experiments conducted after a duration of 48 h that B1 NT‐EPI exhibited superior efficacy against cancer cells compared to free EPI. Additionally, the toxic effects on healthy cells were significantly reduced with B1 NT‐EPI administration compared to free EPI treatment. On the other hand, three groups of nine mice models of bladder cancer were treated with the control group (without drug), free EPI, and B1 NT‐EPI with a concentration of 40 mg/mL of EPI. Each treatment consisted of seven cycles administered at an interval of 5 days. At the end of the treatment period, luminescence measurement showed that in the group of mice treated with B1 NT‐EPI, eight mice were tumor‐free and one mouse showed minimal luminescence signal. To assess the condition of potential EPI cystitis, the bladder tissue from both groups underwent staining with hematoxylin and eosin. In the group treated with B1 NT‐EPI, the lowest rate of cystitis was observed compared to the other groups. Therefore, B1 NT‐EPI shows great promise as a treatment method for bladder cancer.

**FIGURE 9:**
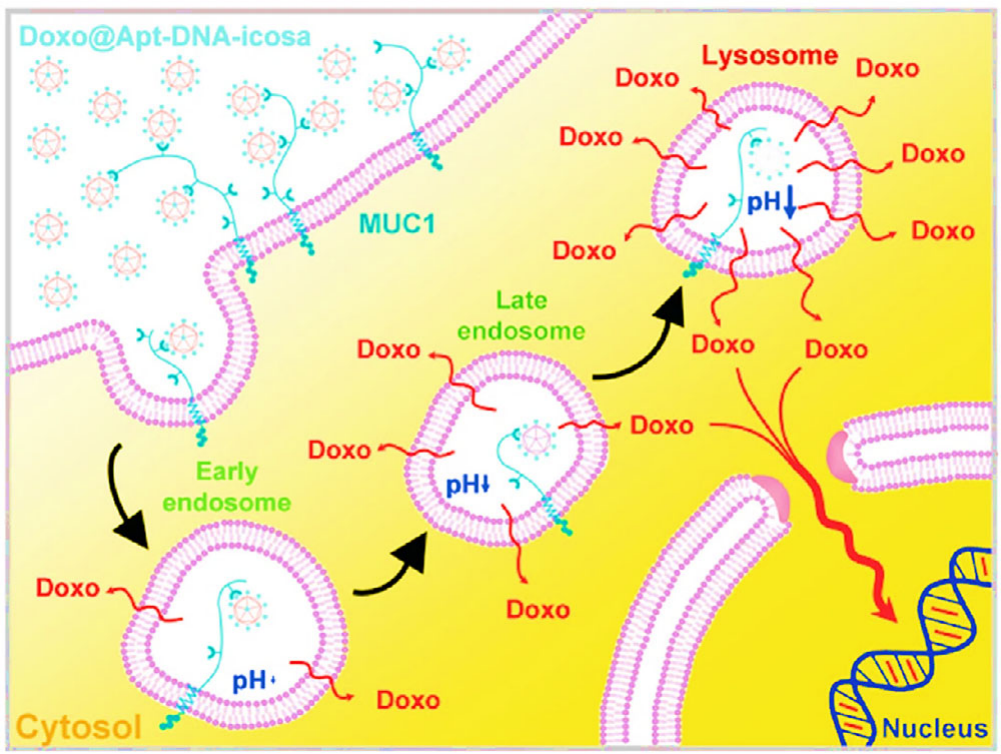
The action pathway of Doxo@Apt‐DNA‐icosa in cells involved cellular uptake by attaching to MUC1 receptors. The ligand‐bound receptor triggered a cellular response. Under specific pH conditions, doxorubicin (DOX) was released and migrated to the nucleus to affect DNA and trigger cell death. Reprinted by permission from ref., 181 Copyright 2011 American Chemical Society.

The high rigidity of icosahedral DNA nanostructures, along with their high selectivity and capacity for carrying drugs, has led to their recognition as drug nanocarriers for cancer therapy. Chang et al.^181^ fabricated a novel 3D DNA icosahedral nanostructure polyhedral. First, a five‐point‐star shape ssDNA (five ssDNA) including 48 nucleotides was generated that contained a primer sequence to avoid loop formation and non‐specific binding in a single‐step process (Figure 9). In a separate step, an aptamer‐integrated six‐point‐star structure (Apt‐DNA‐icosa) was constructed, in which the aptamer was MUC 1 sequence as VI strand. Forty percent of DOX (1000 µM) was then encapsulated in dsDNA of DNA‐icosa and Apt‐DNA‐icosa, which obtained Doxo@DNA‐icosa and Doxo@Apt‐DNA‐icosa, respectively. The cellular uptake study showed that MCF7 cells (MUC1‐positive) had more internalization from Doxo@Apt‐DNA‐icosa than that from Doxo@DNA‐icosa; however, this factor‐induced no difference in CHO‐K1 cells (MUC1‐negative), demonstrating an internalization facilitated by aptamers and high selectivity of this nanoplatform. The designed nanostructure displayed controllable pH‐sensitive release and cytotoxicity MCF7 cells, and no cytotoxic effect on CHO‐K1 cells, while Doxo@DNA‐icosa and free DOX presented no inhibitory effect. The nanocarrier released DOX after internalization, under the influence of pH, and then, Apt‐DNA‐icosa located in lysosomes.

The fabrication cost is an important factor in designing drug nanocarriers. Ma et al.^182^ developed a cost‐effective targeting nanotherapeutic agent based on tetrahedral framework nucleic acid (tFNA), incorporating the HER2 antagonistic aptamer (HApt) and deruxtecan (Dxd), named HApt‐tFNA@Dxd. They investigated the targeting affinity of HApt‐tFNA@Dxd, tFNA, HApt‐tFNA, and tFNA@Dxd in various gastric cells. In gastric tumor cells HGC‐27 and NCI‐N87, which express high levels of the HER2 receptor, HApt‐tFNA@Dxd, and HApt‐tFNA exhibited higher affinity compared to the other groups. However, in GES‐1 (normal cells) with the lowest HER2 expression, the DNA‐based nanostructures showed much lower affinity. In vitro cytotoxicity assessments, HApt‐tFNA@Dxd and free Dxd exhibited higher toxicity toward NCI‐N87 cells, while free Dxd inhibited GES‐1 cells more than the other treatments. The results also indicated that HApt‐tFNA@Dxd treatment led to a marked increase in Bax and Caspase‐3 protein levels while reduced Bcl‐2 protein levels in NCI‐N87 cells, demonstrating that HApt‐tFNA@Dxd has good targeting ability and antitumor activity with minimal side effects on normal cells.

**FIGURE 10:**
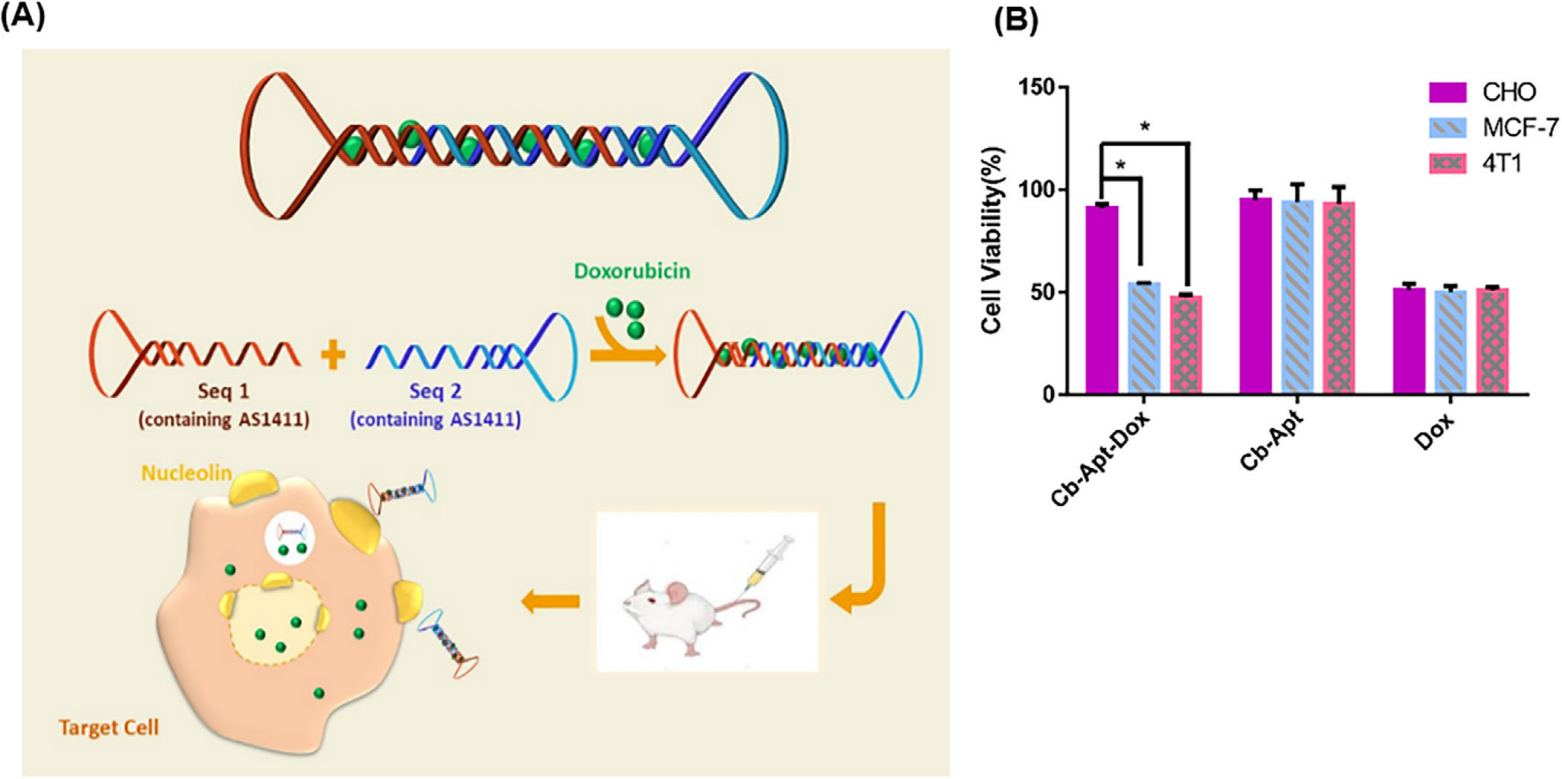
Mechanism and efficacy of Cb‐Apt‒DOX in targeted drug delivery. (A) The design and delivery mechanism of Cb‐Apt‒DOX involved the hybridization of two AS1411 single‐stranded aptamers, resulting in the formation of a double‐stranded structure without free 3′‐ and 5′‐ends. Doxorubicin (DOX) was loaded on the hybridized regions and entered the cell through the aptamer/nucleolin interaction on the cell surface. (B) Evaluation of cell viability using MTT assay after the treatments of MCF7, 4T1, and Chinese hamster ovary (CHO) by Cb‐Apt‒DOX, Cb‐Apt, and free DOX. Reprinted by permission from ref., 183 Copyright 2022 Elsevier.

Resistance to chemotherapy is one of the causes of breast cancer mortality in women. Rahimi et al.^183^ developed a Cb‐Apt system to overcome this problem. The Cb‐Apt strategy consisted of two AS1411 aptamers (specific for nucleolin) on both sides. The absence of free 3ʹ‐ and 5ʹ‐ends in the nanoskeleton carrier protected DNA structural degradation against nucleases and increased the half‐life and stability of the system. The dsDNA in the circular bivalent system acted as a DOX carrier (Figure 10A). After treatment with free DOX, Cb‐Apt, and Cb‐Apt‒DOX, the mortality rate of MCF7 and 4T1 cells (target cells, nucleolin‐positive), as well as Chinese hamster ovary (CHO) cells (non‐target cells, nucleolin‐negative) was assessed using a colorimetric test known as the MTT assay. The respective viability percentages of the various treated 4T1 cells (51%, 93%, and 47%), MCF7 cells (50%, 94%, and 54%), and CHO cells (51%, 95%, and 91%) were obtained, respectively (Figure 10B). According to the results, Cb‐Apt‒DOX had a significant killing effect on nucleolin‐positive cells, while it had no significant effect on nucleolin‐negative cells. This finding highlights the selective binding ability of AS1411 aptamer to nucleolin. The in vivo experiments highlighted that the Cb‐Apt‒DOX system caused a significant reduction in the tumor volume (873.3 ± 21 mm^3^) compared to free DOX (1061.8 ± 23 mm^3^). According to the images obtained from region of interest analysis, 24 h after the injection of Cb‐Apt‒DOX and free DOX into tumor‐bearing mice (4T1 cell line), the accumulation of DOX caused by Cb‐Apt‒DOX in tumor cells was much higher than the other organs (kidney, heart, spleen, lung, and liver). Based on this, the correct targeting power and fewer side effects of Cb‐Apt‒DOX were confirmed compared to free DOX. Cb‐Apt‒DOX with its great stability, high binding affinity, and ability to internalize DOX in target cells is a robust drug carrier with high potential for cancer treatment.

**FIGURE 11:**
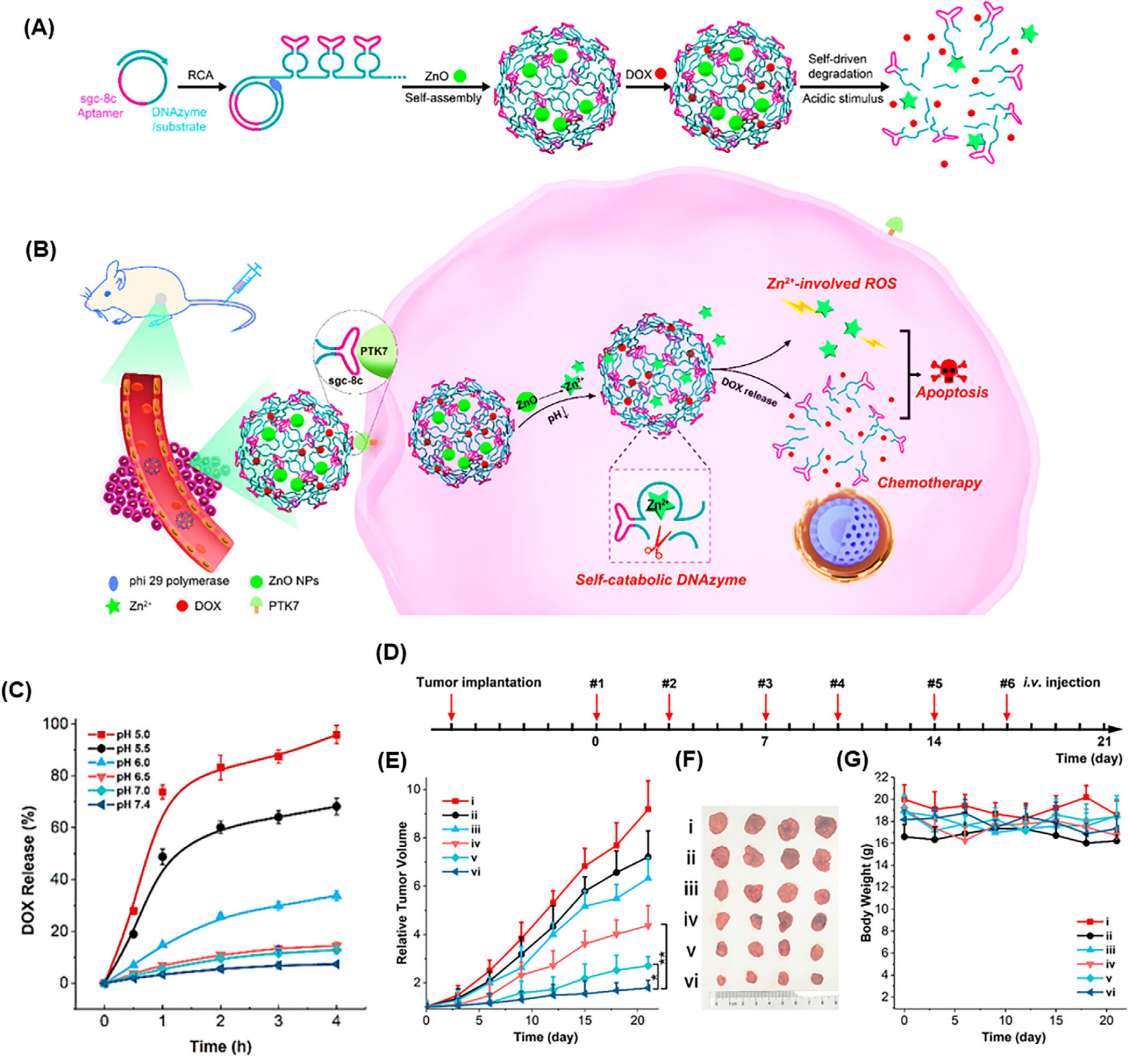
Construction and functional assessment of DOX@RCA‐ZnO‐NSs. (A) Diagram depicting the steps of DOX@RCA‐ZnO‐NSs construction based on rolling circle amplification (RCA) and its acid‐activated breakdown. (B) PTK7 targeting and internalization process that led to doxorubicin (DOX) release and initiated reactive oxygen species (ROS) and cell apoptosis. (C) DOX release behaviors at various pH levels. (D) Timeline of HeLa cell treatment with DOX@RCA‐ZnO‐NSs. (E) Tumor volume graph, and (F) their growth image. Reprinted by permission from ref., 185 Copyright 2019 American Chemical Society.

DNA nanosponges are recognized as outstanding multivalent nanostructures for effectively delivering a variety of therapeutic substances. They can selectively target cancer cells and demonstrate resilience against nuclease degradation, making them ideal carriers for chemotherapy.^184^ These nanostructures can also shield loaded cargo from degradation or premature release in the bloodstream or cellular environment by incorporating specific binding sites or compartments for efficient loading without the risk of leakage. For these reasons, Wang et al.^185^ produced a self‐destructing DNAzyme nanosponge structure through the RCA process (Figure 11A,B). This nanostructure consisted of zinc oxide (ZnO) as the cleavage cofactor, DOX, Sgc8c aptamer, and DNAzyme structure, called DOX@RCA‐ZnO‐NSs. The nanostructure demonstrated prolonged stability in serum, maintaining its nanosponge structure while exhibiting a loading capacity of 5 mmol/g. The drug release strategy was planned as the Sgc8c aptamers precisely targeted protein tyrosine kinase 7 (PTK7) on cancer cells, and then, NSs were taken up by the endocytosis process. In acidic conditions (pH 5.0), ZnO dissolved into Zn^2+^ ions. As DNAzyme cofactors cleaved the NS nanostructure, they caused 95% of DOX release (Figure 11C), simultaneously promoting apoptosis through reactive oxygen species (ROS) involving a p53 pathway. Cytotoxicity was observed in HeLa cells treated with RCA‐ZnO‐NSs, whereas no cytotoxicity was seen with bare RCA NSs, indicating that NS carriers are biocompatible. The DOX@RCA‐ZnO‐NSs provided an apoptosis outcome of 37.4%, the highest amount among all groups. After 21 days of therapy, mice bearing a 50 mm^3^ tumor in the HeLa mouse model showed that the tumor volume increased by only 1.8 times from its initial size when treated with DOX@RCA‐ZnO‐NSs, demonstrating significant anticancer efficacy (Figure 11D‒G).

**FIGURE 12:**
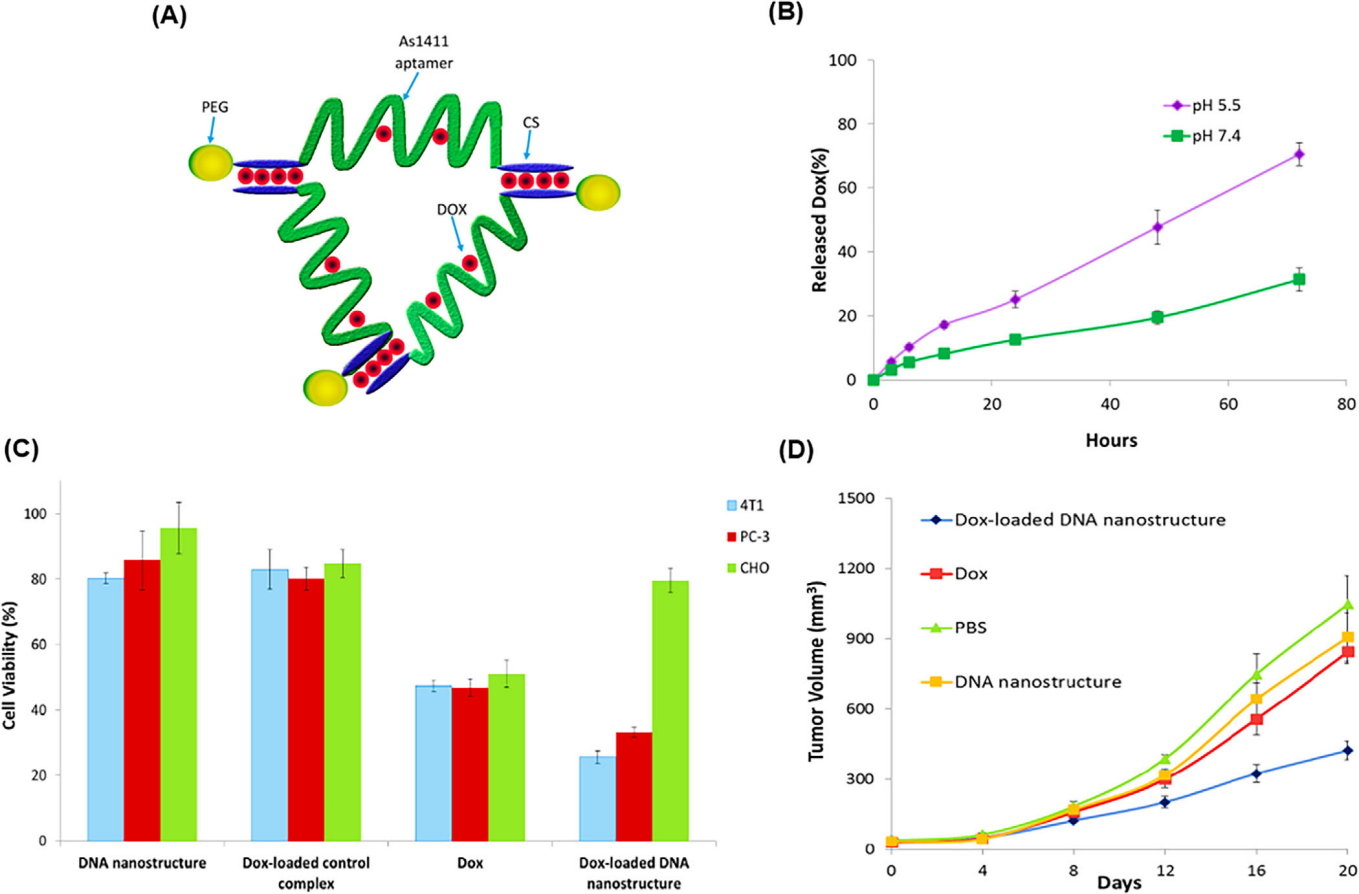
Characterization and therapeutic impact of the three‐way junction (TWJ) pocket DNA nanostructure for doxorubicin (DOX) delivery. (A) The design of the TWJ pocket DNA nanostructure encapsulating DOX included PEGylated three AS1411 aptamer sequences (targeting nucleolin for therapeutic purposes) and entrapped DOX. (B) The DOX release graph in pH 5.5 and 7.4. (C) The results of cell viability in vitro for Chinese hamster ovary (CHO), PC‐3, and 4T1 cells treated with free DOX, DNA nanostructure, DOX‐integrated control complex, and DOX‐integrated DNA nanostructure, within 72 h. (D) In vivo anticancer effects of phosphate buffered saline (PBS), free DOX, DNA nanostructure, and DOX‐integrated DNA nanostructure on 4T1 tumor cells in mice. Reprinted by permission from ref., 188 Copyright 2018 American Chemical Society.

DNA TWJs, as the most basic nanosized supramolecular nanostructures, showed high stability in serum and controlled release in response to different pH levels. Additionally, these nanostructures can specifically target tumor cells with minimal impact on healthy cells, making them suitable for use in drug delivery and cancer therapy.^186^, ^187^ For example, Taghdisi et al.^188^ employed a DOX‐incorporated DNA TWJ nanostructure (Figure 12A) with an optimal loading capacity at a 1:2.5 mol ratio to DOX, maximizing the quenching of DOX fluorescence in fluorometric tests. In vitro release kinetics of DOX encapsulated within the DNA nanostructure were evaluated at pH 5.5 and 7.4, revealing 70% and 32% DOX release over 72 h, respectively (Figure 12B). In vitro cytotoxicity analysis was performed on PC‐3 (human prostate cancer cells), 4T1 (mouse breast cancer cells), and CHO using both the DNA nanostructure loaded with DOX and free DOX (Figure 12C). The cell survival rates were assessed, showing 33.2% for PC‐3 treated with the DNA nanostructure loaded with DOX compared to 46.8% for ones treated with free DOX. For 4T1 cells, the survival rates were 25.6% and 47.4%, respectively. CHO cells exhibited survival rates of 79.6% with the DNA nanostructure and 51% with free DOX. These results demonstrate that the nanosystem has minimal side effects and decreased toxicity for non‐tumor cells, while exhibiting higher cytotoxicity for 4T1 and PC‐3 cells compared to free DOX. In the final in vivo assessment, the volume of the 4T1 tumors was 424 mm^3^ when treated with the DNA nanostructure loaded with DOX, significantly lower than 845 mm^3^ for unencapsulated DOX (Figure 12D). These results suggest that the DNA TWJ nanostructure has a substantial effect on inhibiting tumor cell growth.

#### 3.1.2 Combination therapy

The most common chemotherapy drugs are DOX,^189^ EPI,^190^ and paclitaxel (PTX).^191^ However, the prescription of single‐drug regimens (traditional method) is not effective in treating patients. Recent studies have shown that drug combination cancer therapy (DCCT) is effective in treating and overcoming mechanisms of drug resistance,^192^, ^193^ due to the simultaneous targeting of several agents related to tumor growth. Also, many cancers can be detected in advanced stages, at which time conventional treatments are usually not effective,^194^ even after chemotherapy, the risk of recurrence is very worrying in many cancers such as breast,^173^ bladder,^195^ cervix,^196^ pancreas,^197^ and ovary.^198^ An engineered DNA nanostructure can overcome many of these problems with the efficiency of the unique properties of aptamer science and combination with chemotherapy agents. Significant progress has been made in cancer treatment, aiming to achieve drug combination therapy and intelligent simultaneous targeting at specific locations. The advancements in this field are briefly outlined below. Also, the integration of tumor‐targeted treatment approaches in a single treatment system effectively targets different aspects of a cancer cell and provides more comprehensive and impressive anticancer effects than monotherapy. In addition to reducing drug‐related toxicity and complications, it leads to the reduction of multidrug resistance through various mechanisms and ultimately improves overall patient outcomes.^199^, ^200^ A combination of different treatment approaches, such as radiation/chemotherapy,^201^ immunotherapy/chemotherapy,^202^ radiotherapy/immunotherapy,^203^ and gene therapy/chemotherapy,^204^ have been used for tumor suppression. For example, phototherapy together with chemotherapy synergistically has shown promising antitumor effects for chemotherapy‐resistant and radioactive cancer cells.^199^ In cancer treatment, significant progress has been made to achieve effective combinatorial strategies based on DNA nanostructures to replace conventional treatment regimens with intelligent simultaneous targeting at specific sites, reducing side effects and overall treatment costs. The developments in this area are briefly described below.

**FIGURE 13:**
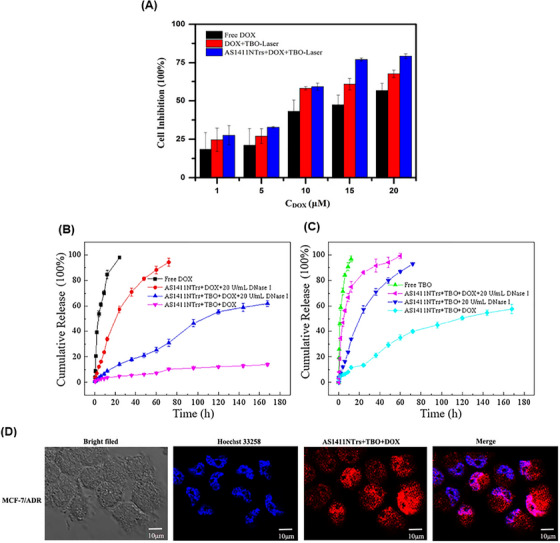
Efficacy and release profiles of doxorubicin (DOX) and toluidine blue O (TBO) in MCF7/ADR cells using AS1411NTrs. (A) Inhibition of MCF7/ADR cells after treatment with the different concentrations of free DOX, DOX with TBO under laser irradiation, and AS1411NTrs loaded by DOX and TBO under laser radiation. Comparison of cumulative release profiles for (B) DOX (substance 1) and (C) TBO (substance 2) in four groups, free DOX solution, AS1411NTrs complexed with substance 1 and 20 U/mL DNase I, AS1411NTrs complexed with substance 2 and 20 U/mL DNase I, and AS1411NTrs complex with substances 1 and 2. (D) After treating MCF7/ADR cells with the AS1411NTrs complex containing substances 1 and 2, confocal laser scanning microscopy (CLSM) imaging revealed the production of blue and red fluorescence, indicating the presence of Hoechst 33258 and DOX, respectively. Reprinted by permission from ref., 205 Copyright 2020 Elsevier.

Multidrug resistance is one of the important causes of the ineffectiveness of chemotherapy. Wu et al.^205^ proposed a novel approach that combines nanotechnology, chemotherapy, and phototherapy to overcome multidrug resistance. In this study, toluidine blue O (TBO) and DOX were used as phototherapy and chemotherapy drugs, respectively, with high potential to overcome MDR. AS1411NTrs formed via aptamer‐triggered HCR (AS1411‐P1) with hairpin monomers H1 and H2, which provided the regions for loading TBO and DOX. According to the results of the differential scanning calorimetry study, the addition of TBO and then, DOX to AS1411NTrs increased the thermal stability. Also, the thermal stability for AS1411NTrs was approximately 70°C, which increased to 77°C after TBO binding. Also, isothermal titration calorimetry analysis showed that TBO binding to AS1411NTrs was driven by large positive ΔΔ*S*° and negative ΔΔ*H*°. According to the K_4_[Fe(CN)_6_] fluorescence quenching study, it was found that TBO interacted with AS1411NTrs through intercalative binding. Additionally, it was observed that the binding site of TBO and DOX was the same. However, DOX exhibited a stronger binding tendency compared to TBO. This indicates that DOX has a higher affinity for the binding site on AS1411NTrs compared to TBO. The AS1411NTrs drugs increased the cell inhibition of MCF7/ADR cells compared to free drugs under laser irradiation (Figure 13A). The release of DOX and TBO from their complex with AS1411NTrs was done slowly within 27 h in phosphate buffered saline (PBS buffer) (pH 7.4). Although 20 U/mL DNase I facilitated the drug release in both conditions, the release of DOX was slower than that of TBO, due to its stronger binding (Figure 13B,C). After treating MCF7/ADR cells with AS1411NTrs + TBO + DOX, the amount of drug accumulation in the cell was evaluated using DOX fluorescence property and confocal laser scanning microscopy (CLSM) imaging. According to the provided images, there was a notable presence of fluorescence in the nucleus rather than the cytoplasm when examining the recovered DOX. This observation serves as evidence for AS1411NTrs' capability to transport drugs effectively into the nucleus (Figure 13D). This strategy with proper targeting of AS1411NTrs and synergistic effect of DOX and TBO can be used as a suitable option to overcome MDR and improve the treatment process.

**FIGURE 14:**
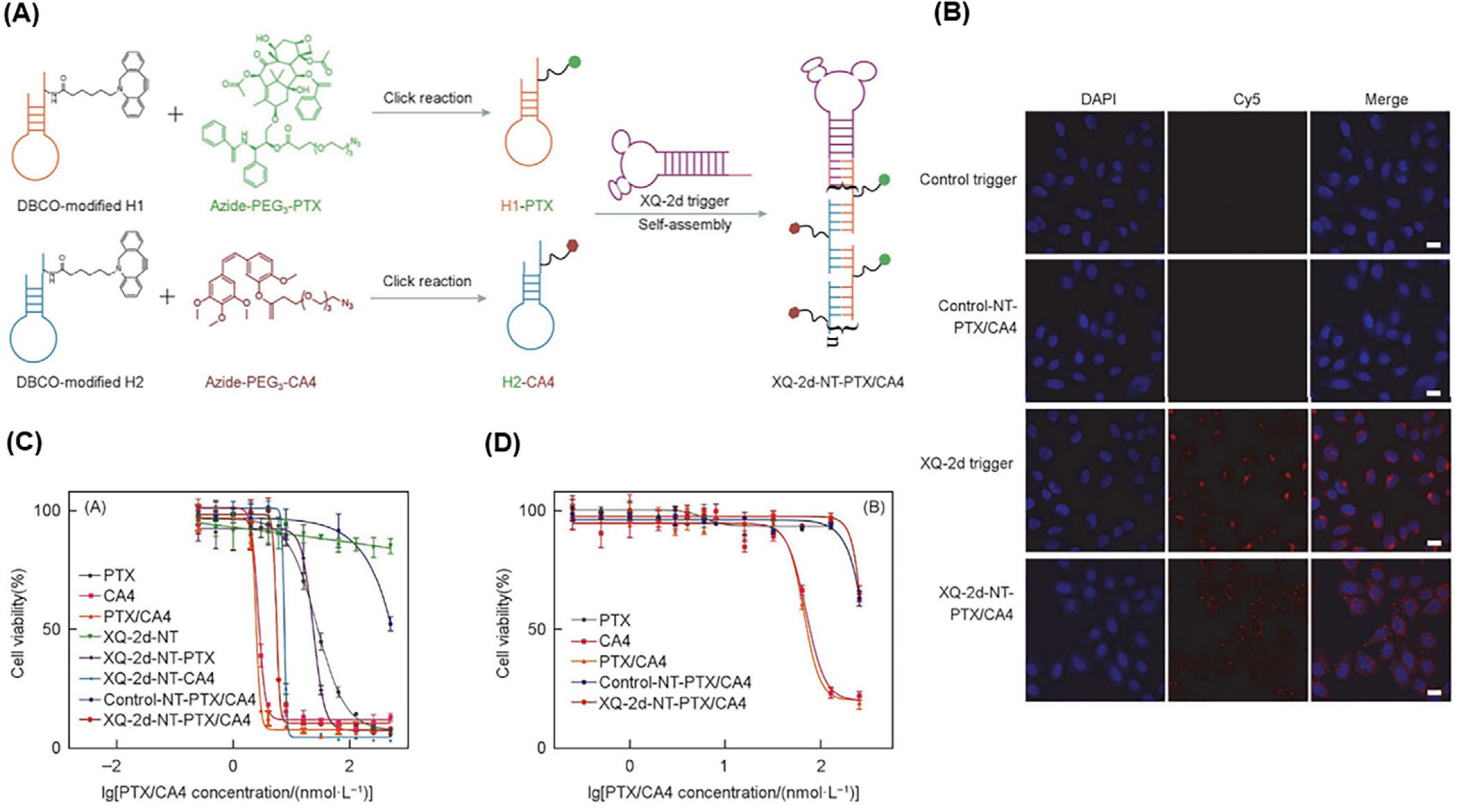
Design strategy and targeted evaluation of XQ‐2d‐NT‐PTX/CA4 for enhanced cancer therapy. (A) Design and function steps of XQ‐2d‐NT‐PTX/CA4. First, two dibenzocyclooctyne (DBCO)‐modified hairpins named H1 and H2 were conjugated to PTX and CA4 through click reaction (H2‐PTX and H2‐CA4); and they were assembled in a DNA nanotrain structure by the hybridization chain reaction (HCR) reaction including XQ‐2d aptamer trigger. (B) Comparison of the binding affinity of XQ‐2d‐NT‐PTX/CA4 with the control group, nanotrain without trigger (NT‐PTX/CA4), and XQ‐2d trigger. (C) Cytotoxicity of XQ‐2d‐NT‐PTX/CA4 compared to its control counterparts on DU145, (D) and HEK293. Reprinted by permission from ref., 207 Copyright 2022 Springer Nature. PTX, paclitaxel.

In another study, Xu et al.^206^ introduced a new nanotrain to deal with drug resistance in breast cancer stem cells (BCSCs). At first, the trigger probe was connected to CD44 aptamer (TA6), and then, it was hybridized with M1 and M2 building blocks containing AKT inhibitors (AKTin). The aptNTrs (TA6NT‒AKTin‒DOX) formed through the HCR reaction. AKT (protein kinase B) plays a very important role in the development of drug resistance in BCSC by increasing ATP‐binding cassette transporters and inhibiting apoptosis. TA6 bound to CD44 and entered BCSCs through TA6NT‒AKTin‒DOX endocytosis. In the acidic environment, such as within the lysozyme‐containing compartments, the structure of the nanotrains was disrupted. This disruption led to the release of AKTin and DOX in close proximity to the nuclear envelope. AKTin inhibited AKT, and synergistically with DOX, led to inhabitation of cell growth and increase of apoptosis. For BCSCs (MCF7 cell line) treated with TA6NT‒AKTin‒DOX and free DOX, IC_50_ values were 764.6 ± 98.9 nM and 3862.0 ± 507.3 nM, respectively (*p* < 0.01). Also, the rate of apoptosis 24 h after treatment was reported as 51.2 ± 2.0% and 23.5 ± 1.5%, respectively (*p* < 0.01) according to the TUNEL assay. Additionally, it was observed that there was a significantly lower accumulation of TA6NT‒AKTin‒DOX in NIH‐3T3 cells (non‐target cells, CD44^−^) compared to BCSCs (target cells, CD44^+^). At 15 days after treatment with TA6NT‒AKTin‒DOX, tumor‐bearing mice demonstrated a significant reduction in both tumor weight (40.8 ± 1.6%) and tumor growth (64.8 ± 2.8%) compared to those treated with free DOX. These results demonstrate the efficacy of TA6NT‒AKTin‒DOX in suppressing tumor growth in the mice model. TA6NT‒AKTin‒DOX has the ability to protect the drug against enzymatic degradation, a high capability for targeted drug delivery, and lower toxicity for healthy cells. These combined features make TA6NT‒AKTin‒DOX a potential solution for overcoming cancer drug resistance and enhancing the effectiveness of cancer treatments. Introducing a platform with the ability to combine therapeutic regimens with the desired synergistic therapeutic effect can be a big step in developing DCCT. Hence, Huang et al.^207^ proposed drug‐carrying aptNTrs (XQ‐2d‐NT‐PTX/CA4) for DCCT. The proposed nanotrain was constructed by using two hairpins (H1 and H2) conjugated with azide‐functionalized PTX and combretastatin A4 (CA4) drug, respectively. They were attached to the aptamer strand through the aptamer‐triggered HCR (XQ‐2d) (Figure 14A). According to the images obtained from flow cytometry and CLSM, after treating DU145 (target cell, CD71^+^) and HEK293 (non‐target cell, CD71^−^) cells with XQ‐2d‐NT‐PTX/CA4, the nanotrain specifically targeted DU145 cells (Figure 14B). The IC_50_ values for DU145 and HEK293 cells were determined by measuring the reduction of the MTS tetrazolium compound in the culture medium, which is a result of living cell metabolism. This measurement was taken after treatment with XQ‐2d‐NT‐PTX/CA4 at a molar ratio of 1:1 for drugs. The calculated IC_50_ value for DU145 cells was found to be 5.19 nmol/L, while it exceeded 300 nmol/L for HEK293 cells. The results indicate that the presented nanotrain exhibits specific selectivity for tumor cells, as demonstrated in Figure 14C,D. XQ‐2d‐NT‐PTX/CA4, with its ability to reset for most of the required drugs and synergistic therapeutic effect, shows great potential as an inspirational strategy for developing DCCT.

**FIGURE 15:**
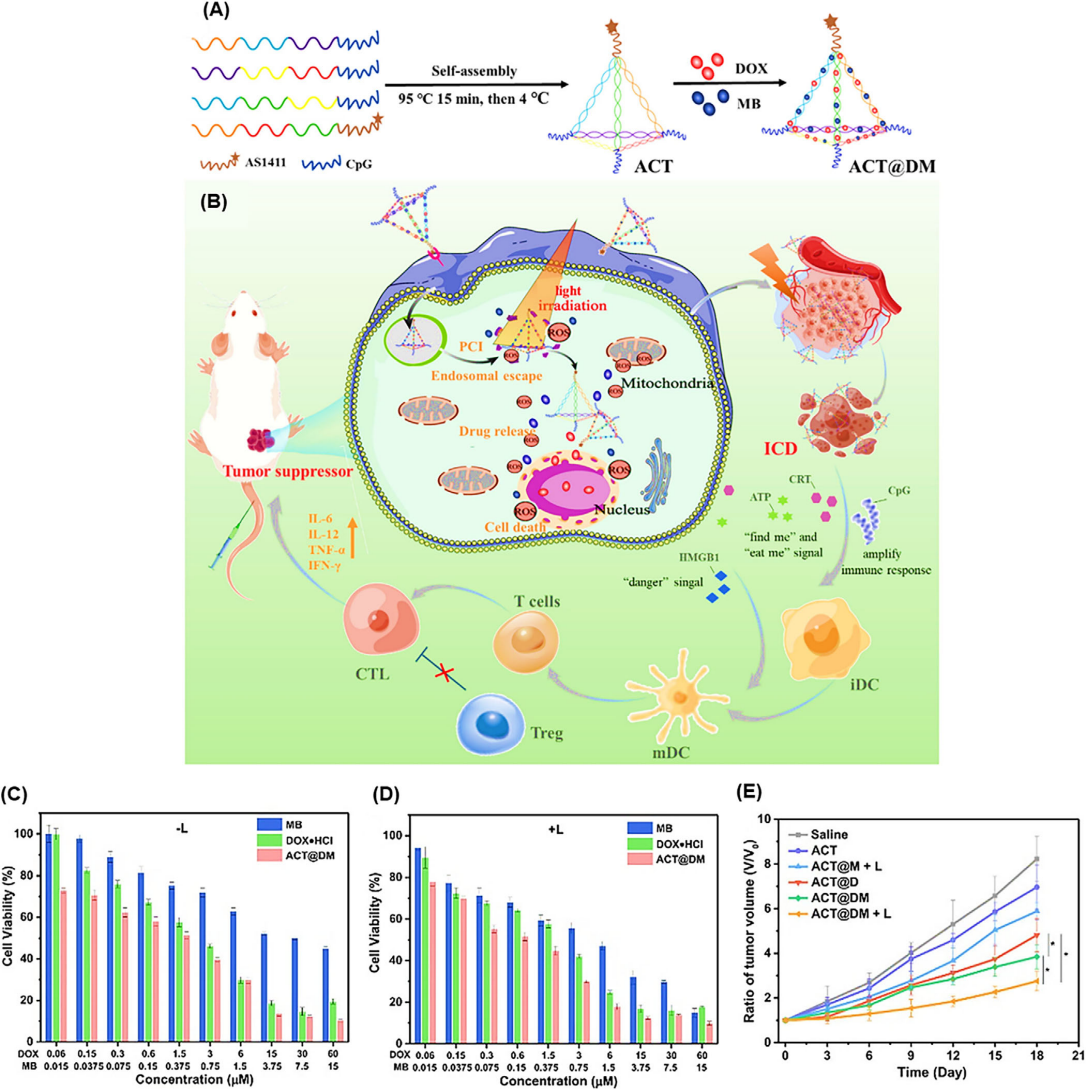
Synthesis and anticancer efficacy of self‐assembling ACT@DM. (A) Synthesis of self‐assemble ACT and adding the drugs resulting in ACT@DM. (B) The anticancer impact of ACT@DM under light irradiation and its performance process. The cell viability of 4T1 cells treated with free doxorubicin (DOX), methylene blue (MB), and ACT@DM at dark (C) and under laser condition (D). (E) The graph of tumor size changes with six groups of treatment. Reprinted by permission from ref., 210 Copyright 2023 Elsevier.

TDNs are self‐assembled structures that have a simple synthesis approach and easily adjustable properties.^208^, ^209^ Due to their applications in cancer therapy, such as carrying antitumor drugs, precisely targeting tumor cells, boosting cellular internalization, and exerting anticancer properties, Yan et al.^210^ designed a novel TDN conjugated with DOX and methylene blue (MB) as a photosensitive agent, called ACT@DM. The TDN included the AS1411 aptamer and CpG (an immunostimulatory adjuvant), providing integrated chemo‐photo‐immune treatment (Figure 15A,B). The loading efficiencies of DOX and MB were 57.3% and 13.0%, respectively, while the encapsulation efficiencies were 67.4% and 35.6%, respectively. The cell viability result of laser irradiation was about 90%, while ACT@DM showed substantial cooperative cytotoxicity under light irradiation compared to without light (Figure 15C,D). In vivo anticancer efficacy on BALB/c mice treated with ACT@DM and laser, ACT@DM, ACT@M and laser, ACT@D, ACT, and saline, and their impact on tumor volume were investigated (Figure 15E). All these groups, except saline (no antitumor efficacy), showed inhibitory effects on tumor growth at different levels. ACT@DM and laser had the best antitumor impact; furthermore, it showed a 60% survival rate of mice within 54 days and no significant side effects, which was markedly better than the other groups. Moreover, ACT@DM and laser showed outstanding performance in elevating the expression of CD86 and CD80, key indicators for DC maturation which enhances T‐cell activation. The ACT@DM and laser treatment led to a 23.6% proportion of CD8^+^ T cells in tumor tissues, a notable reduction in regulatory T cells (Treg) levels, and a CD8^+^/Treg ratio of 12.8%. It also enhanced the levels of tumor necrosis factor‐alpha (TNF‐α), interferon‐gamma (IFN‐γ), interleukin‐6 (IL‐6), and IL‐12p70 in serum, notably in comparison with other treatments, indicating its potential to integrate chemo‐phototherapy with immunotherapy.

**FIGURE 16:**
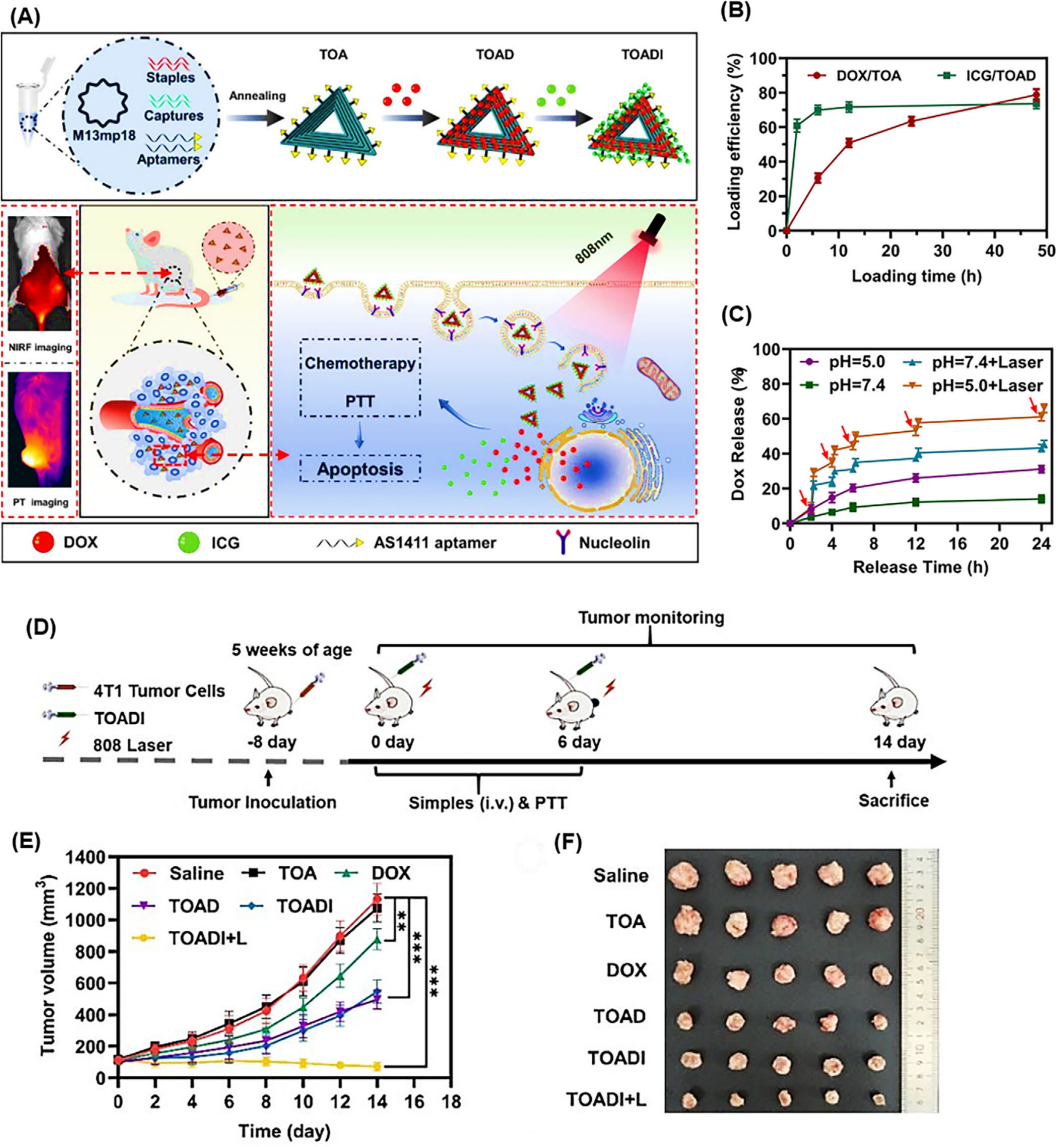
Design and therapeutic evaluation of homogeneous triangular DNA origami. (A) The design process of TOADI in different steps, its in vivo test on 4T1 mouse cells, targeting and internalization procedures, and the final release of doxorubicin (DOX) and indocyanine green (ICG), resulting in chemotherapy and photothermal therapy (PTT). (B) Loading effectiveness of triangular DNA nanostructure over time for ICG and DOX. (C) Release rate of DOX at the different pH levels, with and without laser irradiation. The therapeutic effectiveness of saline, free DOX, TOA, TOAD, TOADI, and TOADI accompanying with laser irradiation on treated mice, along with the process timeline (D) and the final tumor sizes (E), which were observed within 14 days (F). Reprinted by permission from ref., 211 Copyright 2023 BioMed Central.

Triangular DNA nanostructures exhibit high biocompatibility and non‐toxic features in natural environments. Their capability in transporting multiple oligonucleotides enabled Li et al.^211^ to fabricate homogeneous triangular DNA origami modified with the AS1411 aptamer (TOA) to carry DOX and indocyanine green (ICG) for photothermal therapy guided by near‐infrared (NIR) imaging. This resulted in DOX/ICG‐loaded TOA (TOADI), intended for use in breast cancer chemo‐phototherapy (Figure 16A). Initially, the incorporation efficiency of DOX and ICG into TOA was examined. After 12 and 6 h, the effective loading of DOX and ICG exceeded 50% and 70%, respectively (Figure 16B). The release rate of DOX was measured at pH 5.0 and 7.4, showing 30% release in pH 5.0 buffer, notably higher compared to 10% in pH 7.4 (Figure 16C). Furthermore, laser irradiation at pH 5.0 significantly enhanced the release of DOX, achieving 60% release within 24 h. Moreover, laser irradiation boosted the therapeutic effectiveness of TOADI by accelerating DOX release (60% within 24 h), facilitating its delivery to the nucleus of 4T1 cells. The targeting ability of the nanosystem with and without AS1411 aptamers to 4T1 tumor cells was measured by flow cytometry. For this purpose, these cell lines were treated with TOADI and TODI (without aptamer). The former formulation showed significantly higher fluorescence intensities for both DOX (red) and ICG (green) than the latter, proving higher uptake and improved tumor‐targeting capability of the aptamer‐conjugated nanoplatform. Cytotoxicity analysis clarified that TOADI in combination with laser irradiation had lower cell viability than the free DOX, TOADI, and TOAD group. In vivo tumor size evolution in six treated groups within 14 days revealed that TOADI with laser irradiation had the most impact on inhibiting tumor growth, suggesting that combining the photothermal effects of ICG with stable triangular nanostructure carriers (with 84% stability) can enhance the effectiveness of cancer treatments, achieving up to 90% suppression of tumor growth (Figure 16D‒F).

#### 3.1.3 Chemodynamic therapy

**FIGURE 17:**
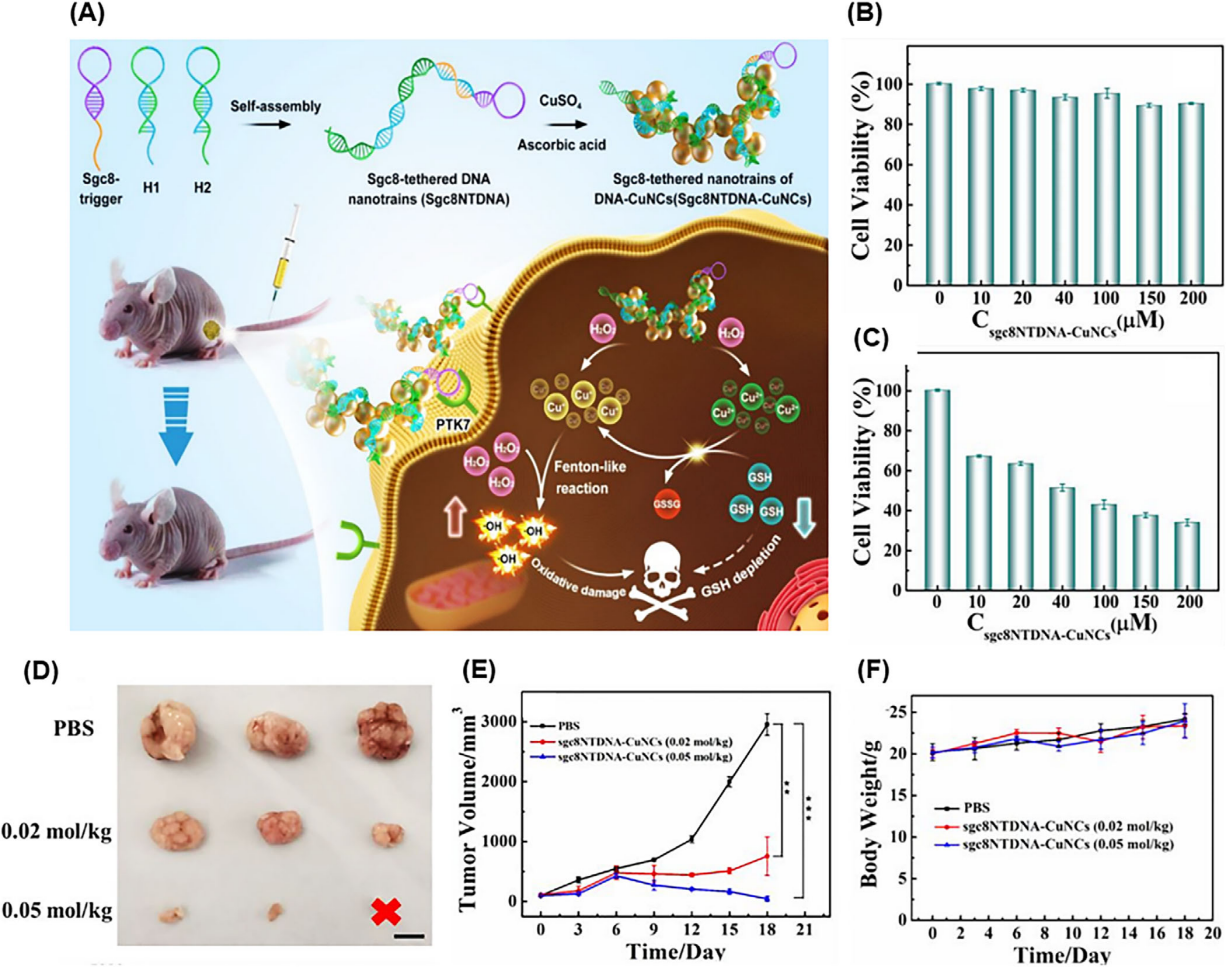
Mechanism and efficacy of sgc8NTDNA‐CuNCs assemblies in cancer therapy. (A) sgc8NTDNA‐CuNCs assemblies entered the HeLa cells by binding to PTK‐7. DNA‐CuNC nanoparticles were oxidized by H_2_O_2_ and a large number of Cu^2+^ and Cu^+^ ions was produced. The Fenton‐like reaction of Cu^+^/H_2_O_2_ and the redox reaction between glutathione (GSH) and Cu^2+^ increased reactive oxygen species (ROS) and decreased the antioxidant property in the cells, which led to increased chemodynamic therapy (CDT) efficiency. (B) Comparison of cell viability after treatment sgc8NTDNA‐CuNCs in HL‐7702 cells (C) and HeLa cells. (D) Comparison of tumor size, (E) tumor volume, and (F) weight changes in HeLa tumor‐bearing mice, 18 days after treatment with phosphate buffered saline (PBS) and two different concentrations of sgc8NTDNA‐CuNCs. Reprinted with permission from ref., 212 Copyright 2022 American Chemical Society.

Most CDT methods have low therapeutic efficacy due to poor targeting. Li et al.^212^ proposed a nanotrain containing copper nanoclusters (sgc8NTDNA‐CuNCs) to overcome this problem in human cervical carcinoma (HeLa). Aptamer sgc8 (PTK‐7 specific) was hybridized to the H1 and H2 sequences as the scaffold for loading CuNCs that achieved the nanotrain structure by the HCR technique. The synthesized sgc8NTDNA‐CuNCs entered the cancer cells; afterward, intracellular hydrogen peroxide (H_2_O_2_) oxidized DNA‒CuNCs and a large amount of Cu^2+^ and Cu^+^ was produced. Through the Fenton‐like reaction of Cu^+^/H_2_O_2_ in the tumor microenvironment (TME) with acidic pH, ROS were produced. Intracellular glutathione (GSH) and Cu^2+^ reduced the antioxidant ability of cancer cells through a redox reaction. The further production of Cu^+^ increased the Fenton‐like reaction of Cu^+^/H_2_O_2_ (Figure 17A). According to the results of MTT assay, the Fenton‐like reaction cascade of Cu^+^/H_2_O_2_ and redox reduction of GSH increased the efficiency of CDT and ability of sgc8NTDNA‐CuNCs to target cells (HeLa) and decreased survival in these cells compared to non‐target cells (HL‐7702) (Figure 17B,C). Eighteen days after the treatment of tumor‐bearing mice with PBS and 0.05 mol/kg of sgc8NTDNA‐CuNC, the tumor size increased from ∼100 to ∼3000 mm^3^ and decreased from ∼100 to ∼40 mm^3^, respectively, and even tumor removal was observed (Figure 17D). Also, sgc8NTDNA‐CuNC significantly reduced tumor volume with minimal effect on mouse weight (Figure 17E,F). The proposed nanotrain can be a promising and biocompatible method to improve CDT by applying suitable aptamer in all types of cancer.

#### 3.1.4 Cytotoxic proteins

**FIGURE 18:**
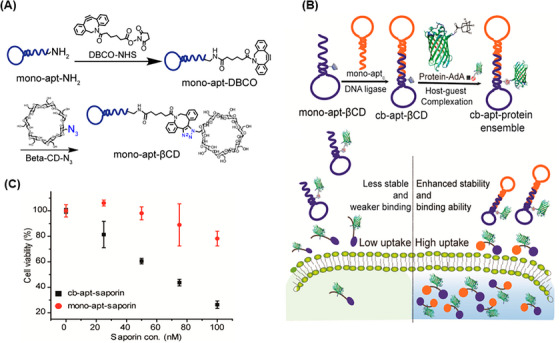
Enhanced therapeutic potential of the circular bivalent aptamer (Cb‐Apt)‒saporin complex in targeting HeLa cells. (A) Illustration of the Cb‐Apt‒*β*CD manufacturing steps. In the first step, sgc8 aptamer (specific to PTK‐7) was modified by an amine group (mono‐apt‒NH_2_) that could react with dibenzocyclooctyne‐sulfo‐N‐hydroxysuccinimidyl ester (DBCO‒NHS) to improve the binding of *β*‐cyclodextrin (*β*CD) to the sgc8 aptamer. Then, mono‐apt‒DBCO and 6‐monoazido‐6‐monodeoxy‒*β*CD were mixed at room temperature and *β*CD‐conjugated mono‐apt‒*β*CD was obtained by click conjugation. (B) In the next step, it was hybridized with the complementary aptamer that formed Cb‐Apt‒*β*CD. The adamantane‐modified protein formed a host‒guest complex with the *β*CD region through supramolecular interaction. (C) Comparison of HeLa cells viability after the treatment with Cb‐Apt‒saporin complex and mono‐apt‒saporin. Reprinted with permission from ref., 214 Copyright 2018 American Chemical Society.

In addition to drugs, much attention has been paid to cytotoxic proteins to overcome cancer. Cytotoxic proteins have high anticancer activities, and due to their large size, they can effectively remain inside the target cells and prevent the exit of therapeutic agents through multidrug resistance transporters. One of the important challenges in the clinical translation of these proteins is their ineffective delivery to tumor cells.^213^ Jiang et al.^214^ proposed a supramolecular complex based on Cb‐Apt capable of host‒guest interaction with a therapeutic agent for targeted delivery into cells. To construct the Cb‐Apt‒*β*CD, sgc8 aptamer was modified by primary amine and dibenzocyclooctyne‐sulfo‐N‐hydroxysuccinimidyl ester (DBCO‒NHS); then, *β*‐cyclodextrin (*β*CD) conjugated with mono‐apt‒DBCO was obtained through click conjugation (Figure 18A). Adamantane (AdA), as a hydrophobic moiety, was used to form the host‒guest inclusion complex with *β*CD. Cb‐Apt‒*β*CD showed a high cellular uptake ratio to mono‐apt‒*β*CD along with efficient delivery of GFP‐AdA to HeLa (PTK‐7, positive) cells (Figure 18B). For further study, a cytotoxic protein called saporin was loaded in *β*CD‐conjugated aptamer. After treating HeLa cells with the Cb‐Apt‒saporin complex, cell viability decreased by 20%, whereas mono‐apt‒saporin showed no significant toxicity. In conclusion, the Cb‐Apt‒*β*CD complex effectively improved the intracellular delivery of saporin (Figure 18C). Also, the anticancer agent AuNHC (N‐heterocyclic carbene [NHC]‐gold(I)) was loaded on Cb‐Apt‒*β*CD by hydrophobic interaction. Cb‐Apt‐AuNHC had fivefold more cytotoxicity than mono‐apt‐AuNHC for CEM (PTK‐7, positive) cells in medium containing serum. These results prove that Cb‐Apt‒*β*CD is able to increase stability in serum and can be used as a unique complex for targeted drug loading and delivery for therapeutic platforms.

### 3.2 Oligonucleotide therapy

Oligonucleotides are short synthetic nucleic acid fragments that prevent gene expression by interfering with translation through various mechanisms.^215^ Various oligonucleotides were introduced with the aim of tumor suppression, such as small interfering RNA (siRNA), immunostimulatory CpG oligonucleotides, miRNA oligonucleotides, and antisense oligonucleotides (ASOs).^216^, ^217^, ^218^, ^219^, ^220^ However, the success of these therapeutic approaches mainly depends on the efficiency of gene transfer carriers.^216^, ^217^ One of the common methods for gene transfer is viral carriers, but immunogenicity and unpredictable consequences are the reasons for their failure.^221^ Also, most of these carriers transfer therapeutic agents to the cytoplasm of tumor cells, while only a small percentage of these drugs are transferred to the nucleus. This can result in a decrease in the therapeutic effect and even non‐drug activation.^222^, ^223^, ^224^, ^225^ Also, many common anticancer drugs, such as DOX, act on nuclear DNA and cause the death of cancer cells by disrupting replication.^226^ Therefore, a reliable carrier to transfer the medication to the cell nucleus can shorten the treatment process and reduce the drug dosage and side effects. DNA nanostructure has been investigated as a suitable option with promising efficacy and significant safety for gene transfer due to their high loading capacity of gene silencing agents, stability, and flexibility.^227^, ^228^, ^229^ The combination of gene therapy and chemotherapy with specific targeting will increase the treatment efficiency in cancer patients. We will report on recent developments in this field.

**FIGURE 19:**
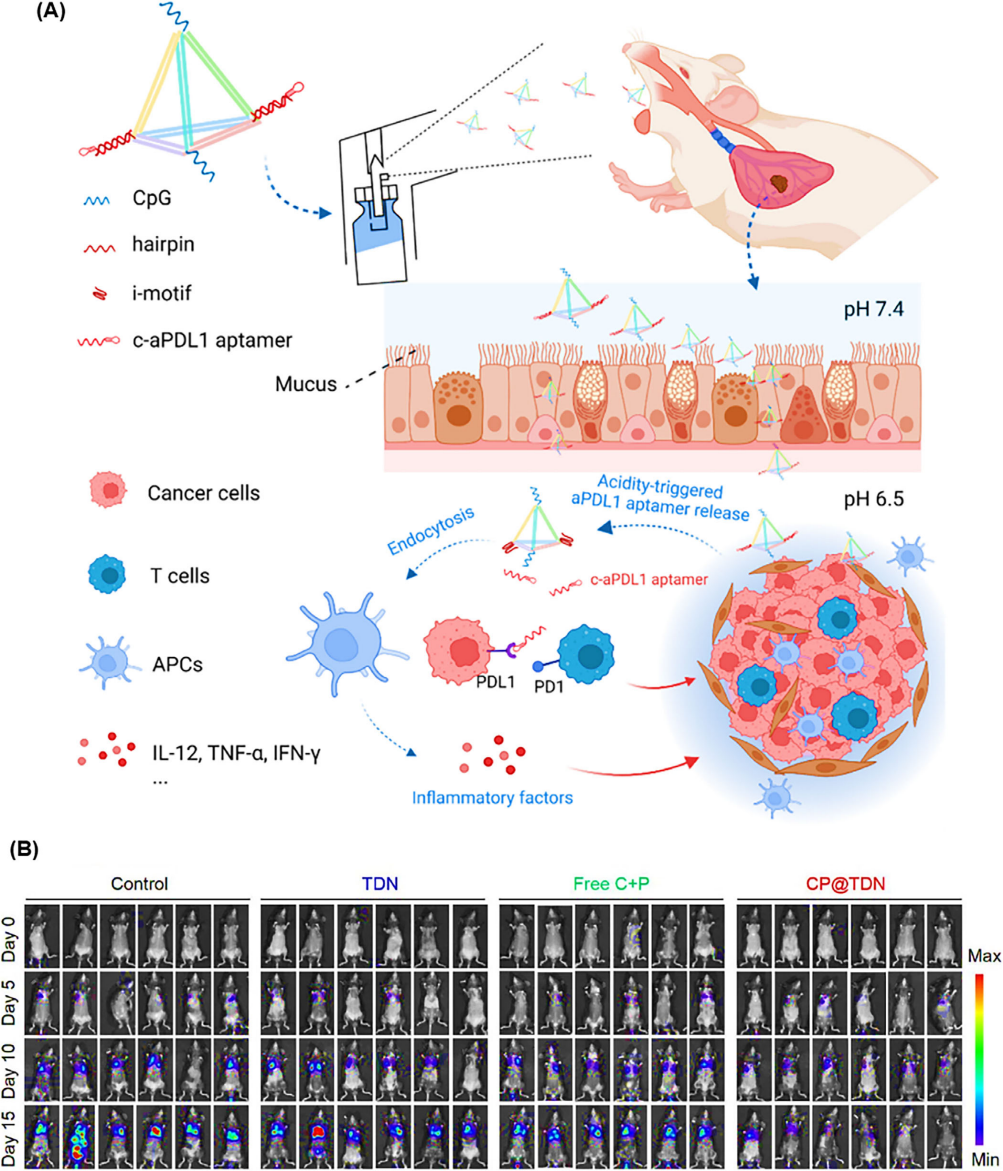
Overview of the CP@TDN structure and its therapeutic effects on lung tumors. (A) Schematic diagram of the CP@TDN structure and its mechanism of action on lung tumor cells. (B) Results of bioluminescence imaging for four treated groups of mice. Reprinted with permission from ref., 231 Copyright 2023 Elsevier. TDN, tetrahedral DNA nanostructure.

The high permeability capacity of TDNs, due to their small size, makes them an appropriate nanocarrier for cancers where penetration is a challenge.^15^, ^230^ Given that, Fan et al.^231^ constructed a TDN‐based nanocarrier containing an immune‐regulating CpG oligonucleotide and a programmed death‐ligand 1 (PD‐L1) DNA aptamer, referred to as CP@TDN (Figure 19A). The TDN, with an approximate edge length of 6.0 nm, was designed to inhibit lung tumor cell growth through inhalation, allowing it to penetrate the pulmonary mucosal barrier and significantly increase accumulation within lung tumor cells. These TDN delivery platforms were developed for the extracellular release of apt‐PD‐L1 in response to weakly acidic tumor conditions, blocking PD‐L1 receptors on tumor cells while enabling CpG uptake by immune cells to stimulate an immune response. In vivo antitumor effects were evaluated using B16F10 cells in four groups of treated mice: CP@TDN, free both C and P, TDN, and saline. Unlike the other three groups, the CP@TDN‐treated group displayed significant tumor growth suppression by elevating the levels of pro‐inflammatory cytokines, including IFN‐γ, TNF‐α, IL‐6, and IL‐12 in the serum, as shown in Figure 19B. Subsequently, the immune response was assessed, revealing that CP@TDN treatment increased CD45^+^ leukocytes and maturation of dendritic cells. The proportion of CD3^+^ T cells in tumors was 3.85% for CP@TDN, compared to 1.42% for TDN and 2.68% for free C and P. The percentage of CD8^+^ T cells was also higher in the CP@TDN group at 1.43%, compared to 0.96% for free C and P, 0.49% for TDN, and 0.29% for the untreated group. These results suggest that CP@TDN can inhibit pulmonary tumor growth by eliciting a robust antitumor immune response.

#### 3.2.1 Gene therapy

**FIGURE 20:**
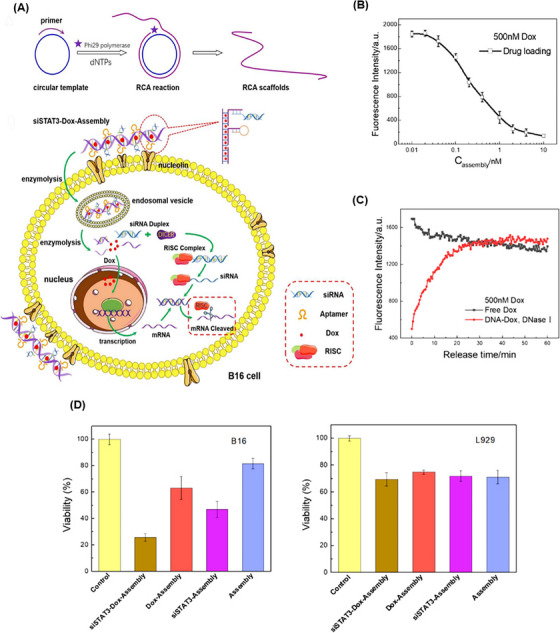
Construction and mechanism of action of the siSTAT3‒DOX nanostructure in melanoma therapy. (A) Design and mechanism of siSTAT3‒DOX‒Assembly. During the rolling circle amplification (RCA) method, a long scaffold strand comprising three short chains, including complementary siRNA, nucleolin‐specific aptamer sequence, and a doxorubicin (DOX) loading site, was formed using a circular DNA template and a special polymerase. By binding to nucleolin, it entered the cell and released siRNA. With activation of the RNA‐induced silencing complex (RISC) complex, it cleaved the target mRNA and DOX was also released in this place. (B) Fluorescence intensity resulting from DOX loaded in siSTAT3‐Assembly. (C) The release curve resulting from the fluorescence changes loaded DOX in siSTAT3‒DOX‒Assembly after adding endonuclozyme. (D) Comparison of cytotoxicity after treatments with siSTAT3‒DOX‒Assembly, individual components of the nanotrain complex, and the control group between target cells B16 (left) and non‐target cells L929 (right). Reprinted with permission from ref., 232 Copyright 2023 American Chemical Society.

Dual delivery of chemotherapy and genetic drugs has been considered due to its high potential in inhibiting cancer cells. In this case, Zhang et al.^232^ presented an RCA‐based nanotrain to inhibit the mouse melanoma cell line (B16) (Figure 20A). siSTAT3‒DOX‒Assembly formed using the RCA technique as a product of three short strands, including a complementary strand of siRNA (target STAT3 of mRNA), aptamer sequence (specific for target nucleolin), and complementary sequence of RCA strand for DOX loading. siSTAT3‒DOX‒Assembly could enter B16 melanoma cells by binding to cell surface nucleolins through endocytosis. The siRNA was released and then, the RNA‐induced silencing complex formed and activated, which degraded the target mRNA and inhibited its translation. At the same time, DOX was released and exerted its therapeutic effect. Also, the decrease in fluorescence intensity resulting from the DOX loading in DNA‒DOX complexes proved the correct placement of DOX in the DNA double helix. Upon addition of endonuclease to siSTAT3‒DOX‒Assembly, DOX was released within 30 min, and a fluorescence level equal to free DOX was achieved (Figure 20B,C). siSTAT3‒DOX‒Assembly possessed a significant effect in inhibiting cell metastasis and cell growth in target cells compared to non‐target cells and free DOX (Figure 20D). This system, with its high potential in targeting and therapy, shows promise for the treatment of specific types of cancer.

**FIGURE 21:**
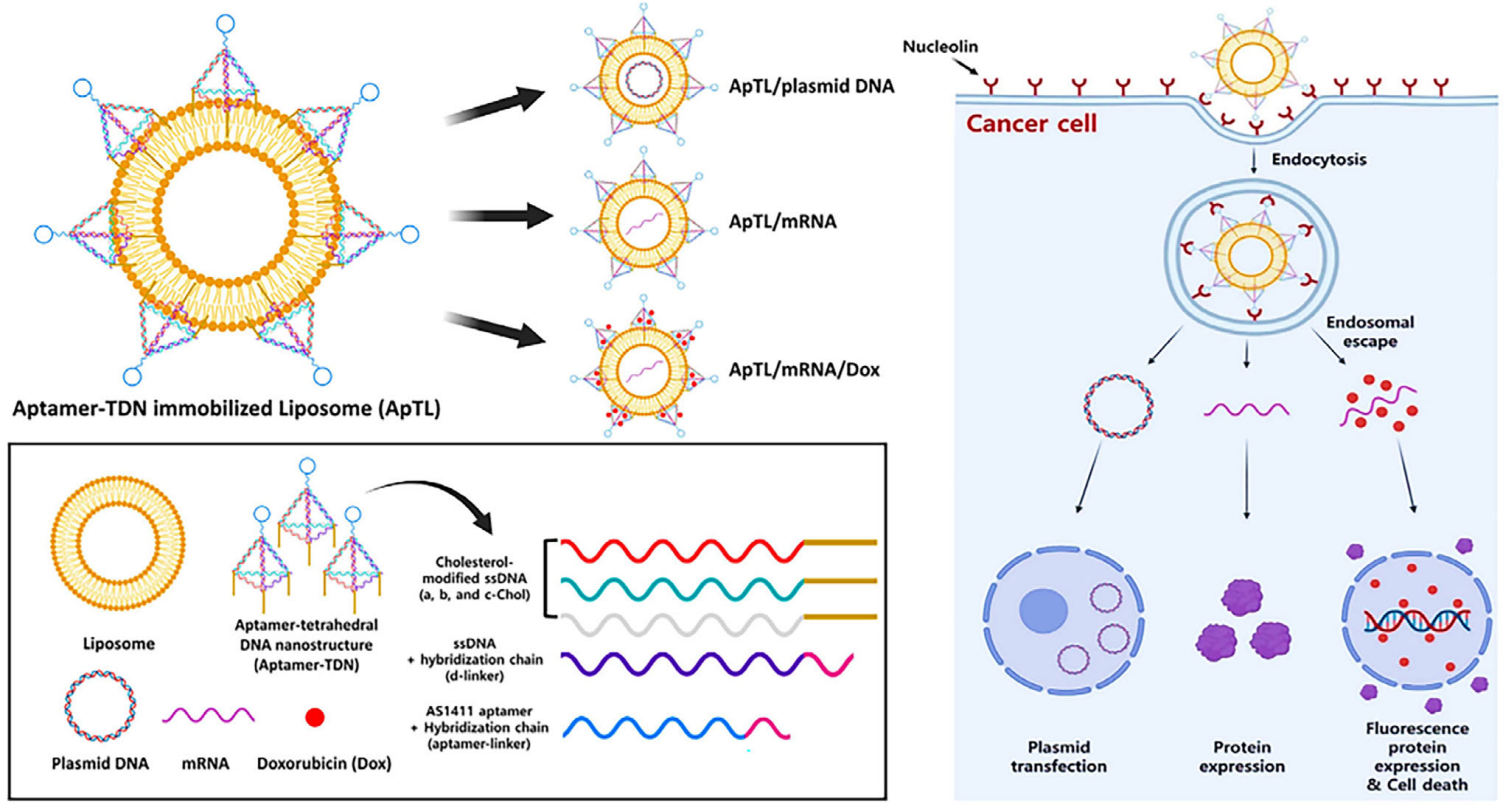
The schematic representation of different ApTL structures (with mRNA, plasmid, and mRNA with doxorubicin [DOX]) and their internalization in tumor cells. Reprinted with permission from ref., 233 Copyright 2023 John Wiley & Sons, Ltd.

To protect nucleic acid structures from degradation and safely deliver them to target cells, TDN can be used in combination with liposomes. For example, Lim and Hwang^233^ introduced a new TDN‐based nanocarrier, a liposome with immobilized TDN modified by an aptamer, containing the AS1411 aptamer (called ApTL) to carry mRNA and plasmid. As shown in Figure 21, aptamer‐TDN was fabricated with ssDNA modification using three cholesterol variants (Chol a, b, and c) and linked to the AS1411 aptamer with a d‐linker. Its function was to target nucleolin on the surface of tumor cells, avoid binding to non‐specific targets, and ensure long‐term blood circulation due to its negative charge. The ApTL‐treated normal cells displayed 97.4% viability, indicating that it is a safe carrier. In a cytotoxicity assessment, ApTL/mEGFP/DOX (including EGFP mRNA) treated cells (EGFP‐positive) within 48 h revealed 15.4% viability, which was lower than the 38.6% viability for ApTL/mEGFP without DOX. The impact of ApTL/mEGFP might be due to the AS1411 aptamer attachment to nucleolin, which inhibited tumor cell growth by disrupting crucial signaling pathways, making it a potentially safe and specific carrier for targeting tumors.

**FIGURE 22:**
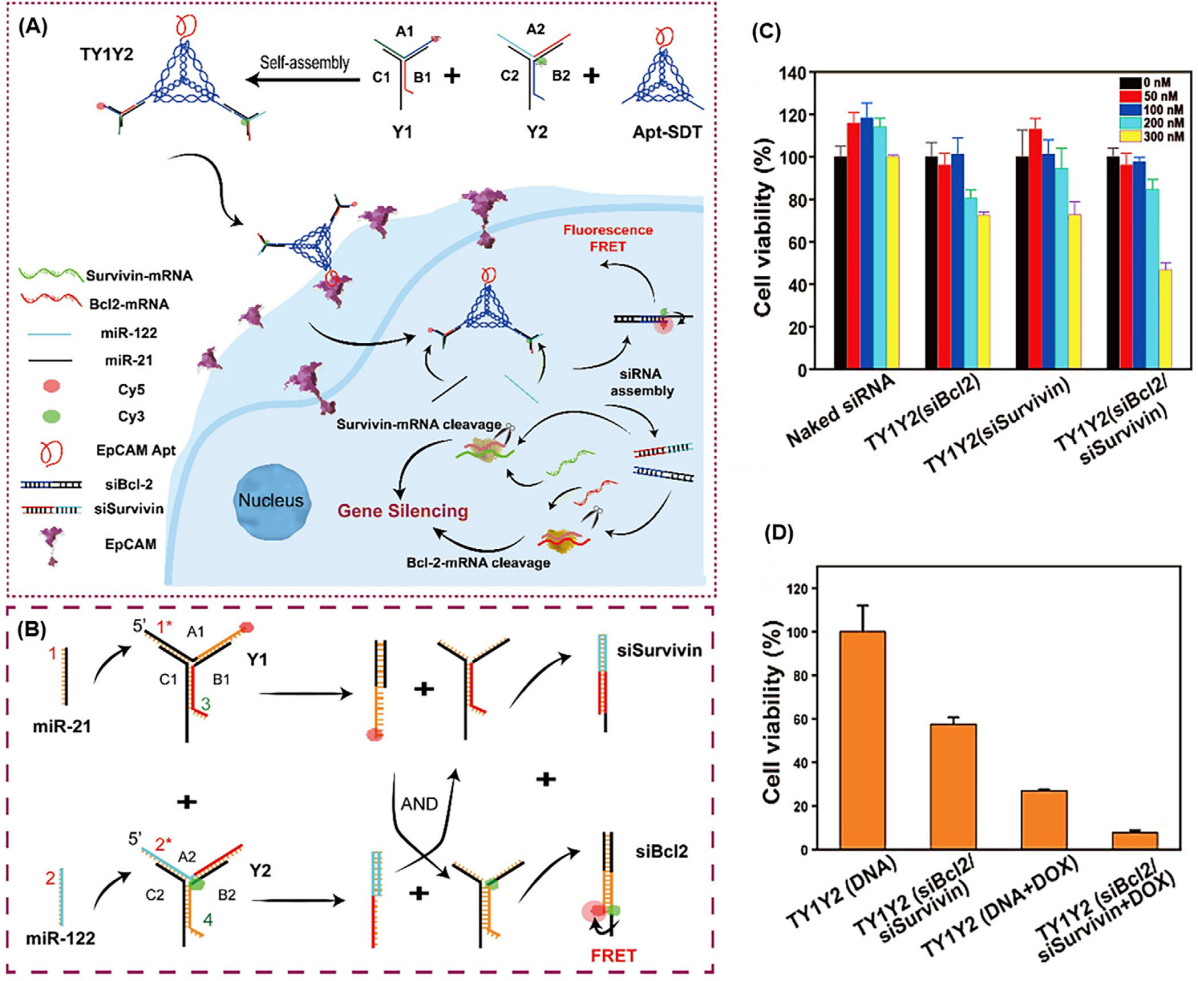
Self‐assembly, cytotoxicity, and cell viability of the TY1Y2 nanostructure for hepatocellular carcinoma treatment. (A) Synthesis procedure of the TY1Y2 structure, mechanism of cell entry, and its effect on cell apoptosis. (B) The process of Y1 and Y2 disassembly by miR‐21 and miR‐122 that results in fluorescence resonance energy transfer (FRET). (C) Cytotoxicity of TY1Y2 (siBcl2/siSurvivin), TY1Y2 (siSurvivin), TY1Y2 (siBcl2), and free siRNA in the treatment of Huh‐7 cells. (D) The cell viability of Huh‐7 cells treated with TY1Y2 (siBcl2/siSurvivin) and doxorubicin (DOX). Reprinted with permission from ref., 235 Copyright 2023 Springer Nature.

TDNs can be used for gene therapy purposes, as well as imaging. They can be combined with some rigid DNA structures to resist DNase enzymes and with specific tumor cell ligands to improve the performance of carrying and targeting, while decreasing off‐target impacts.^234^ Zhong et al.^235^ designed a 3D DNA nanostructure named TY1Y2, consisting of Apt‐SDT (EpCAM‐aptamer functionalized tetrahedral structure) with the ability to bind to specific ligands on tumor cells, and two Y‐shaped DNA, Y1, and Y2, assembled with three ssDNAs (A1, B1, C1, and A2, B2, C2, respectively) (Figure 22A,B). A1 and B2 consisted of the sense and antisense sequences of siBcl2, and A2 and B1 consisted of the antisense and sense sequences of siSurvivin, respectively. After entering the cell, Y1 and Y2 were disassembled in the presence of both miR‐122 and miR‐21, and they reformed into miR‐21/A1/B2 and miR‐122/A2/B1, the siBcl2 and siSurvivin duplex patterns, respectively, inducing fluorescence resonance energy transfer and concurrent dual miRNA imaging. The release of siSurvivin and siBcl2 led to gene silencing and reduced cell proliferation. The apoptosis study for TY1Y2 (siBcl2/siSurvivin) showed coordinated gene suppression of siSurvivin and siBcl2 (Figure 22C). Additionally, the inhibitory effect of TY1Y2 (siBcl2/siSurvivin) combined with DOX was investigated. The results shown in Figure 22D demonstrate that the combination of oncoprotein silencing and chemotherapy can enhance antitumor efficacy.

### 3.3 Bioimaging

Monitoring drug delivery and understanding its therapeutic functions is important to evaluate treatment steps.^236^ Therefore, the design of carriers with targeted drug delivery and simultaneous imaging agents greatly contributes to the treatment process.^237^, ^238^, ^239^ Among the imaging agents, MBEs and the inherent fluorescence of certain drugs are of great importance.^216^ Non‐specific uptake of imaging agents remains a challenge for high‐quality imaging. In aptamer‐linked DNA nanostrands, the aptamer part improves targeting and accumulation in tumor tissue, and box car parts provide the possibility of high‐capacity loading of therapeutic agents and biological imaging with real‐time signaling. It helps us to understand the behavior of drugs in the target cells during the treatment process.^240^, ^241^ Theranostic platforms, such as DNA nanostructures, with extensive features, have the ability to be modified and personalized, which ensures their wide application in the field of dual function of treatment and diagnosis in the future. In the following, we report some recent developments in this direction.

#### 3.3.1 Imaging using Ce6 and anthracyclines

**FIGURE 23:**
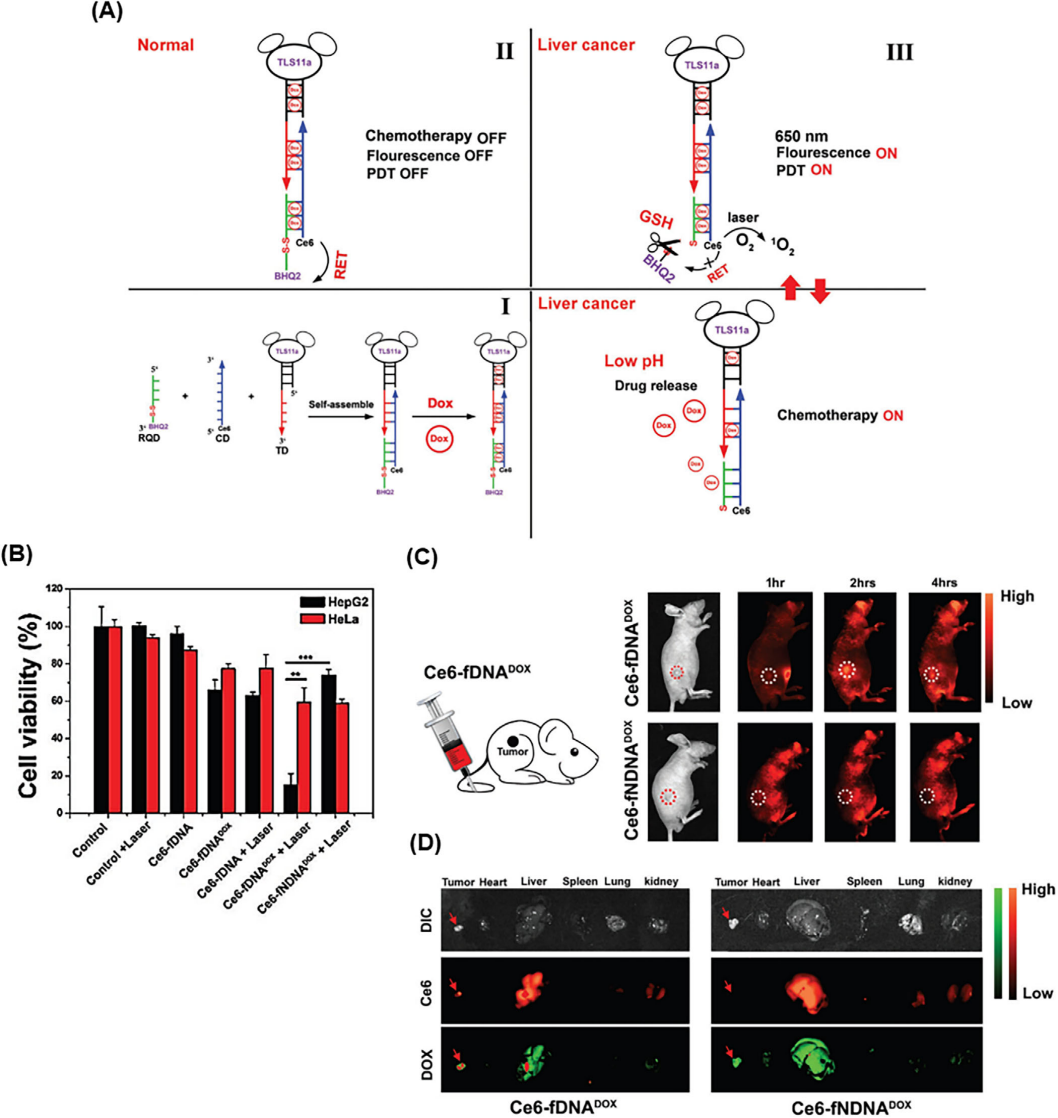
Design and functional analysis of the Ce6‐fDNA nanostructure for enhanced targeted therapy in human liver cancer. (A) Overview of Ce6‐fDNA^DOX^ probe fabrication and performance. (I) Three types of single‐stranded DNA (ssDNA), modified by TLS11a, chlorin e6 (Ce6), and RQ (with BHQ2 quencher), were self‐assembled. Doxorubicin (DOX) was loaded into the double‐stranded regions. (II) In normal cells, the BHQ2 quencher could not be released due to the low level of glutathione (GSH), and the probe was in the “OFF” state. (III) By entering the cancer microenvironment BHQ2 quencher was released due to the high level of GSH, and reactive oxygen species (ROS) was induced from Ce6. At the same time, DOX was released because of low pH; as a result, fluorescence, photodynamic therapy (PDT), and chemotherapy were set to “ON” state. (B) Comparison between the viability of HepG2 cells (target) and HeLa cells (non‐target) after treatment with Ce6‐fNDNA^DOX^, Ce6‐fDNA^DOX^, and Ce6‐fDNA with or without laser irradiation (670 nm). (C) Timelapse fluorescence images of tumor‐bearing mice after treatment with Ce6‐fDNA^DOX^ and Ce6‐fNDNA^DOX^. (D) Optical and fluorescence images of biodistribution of Ce6 and DOX (in red and green, respectively) after treatment with fDNA^DOX^ and Ce6‐fNDNA^DOX^. Reprinted by permission from ref., 242 Copyright 2017 John Wiley & Sons, Ltd.

Zhang et al.^242^ developed a smart Ce6‒DOX‒DNA hybrid by combining photodynamic therapy (PDT), chemotherapy, and real‐time fluorescence imaging against hepatocellular carcinoma cells (Figure 23A). Ce6‒DOX‒DNA hybrid, named as a Ce6‐fDNA^DOX^ probe, was self‐assembled by hybridization of three types of functionalized ssDNA with cell‐targeting aptamer TLS11a (TD), Ce6 labeled ssDNA (CD), and redox‐responsive quencher ssDNA (RQD). To increase the efficiency of fluorescence imaging and simultaneously PDT treatment of cancer cells and reduce side effects, the black hole quencher 2 (BHQ2) group was attached to the end of the RQD chain through a disulfide linkage. In normal cells, due to fluorescence resonance energy transfer, the ability to emit fluorescence and ROS generation from Ce6 was quenched by BHQ2 (“OFF” state of PDT). But in cancer cells, due to the high amount of GSH, the disulfide linkage was broken and the BHQ2 quencher was released. Therefore, the ability of ROS generation and fluorescence, for PDT treatment and NIR imaging, was restored from Ce6 in these cells. On the other hand, the loaded DOX in the double‐strand regions through π‒π stacking was released in response to the low pH of cancer cells. Meanwhile, a synergistic treatment of chemotherapy with the “ON” state of PDT resulted in these cells. The inhibition rate of HepG2 cancer cells after treatment with Ce6‐fDNA with laser irradiation, Ce6‐fDNA^DOX^ without laser irradiation, Ce6‐fDNA^DOX^ with laser irradiation, and Ce6‐fNDNA^DOX^ (without specific aptamer) were 37.9%, 33.9%, 85.1%, and 26.1%, respectively. As a consequence, Ce6‐fDNA^DOX^ together with laser radiation could effectively destroy the target cells. On the contrary, the lowest inhibitory effect was observed on the non‐target cells (HeLa cells) (Figure 23B). In the tumor‐bearing mouse model (HepG2 cell line), a significant Ce6 fluorescence signal was observed at the tumor site after 2 h of Ce6‐fDNA^DOX^ injection, but it gradually decreased with passing time. However, no significant Ce6 fluorescence signal was observed after injection of the Ce6‐fNDNA^DOX^ at the tumor site, which indicates correct targeting of TLS11a aptamer (Figure 23C). Also, the amount of biodistribution of DOX and Ce6 by Ce6‐fDNA^DOX^, compared to Ce6‐fNDNA^DOX^, was higher in the tumor site than in the vital organs, especially the heart and lung (Figure 23D). This purposefully engineered platform with “ON” and “OFF” PDT minimizes side effects and improves treatment performance by combining synergistic chemotherapy.

**FIGURE 24:**
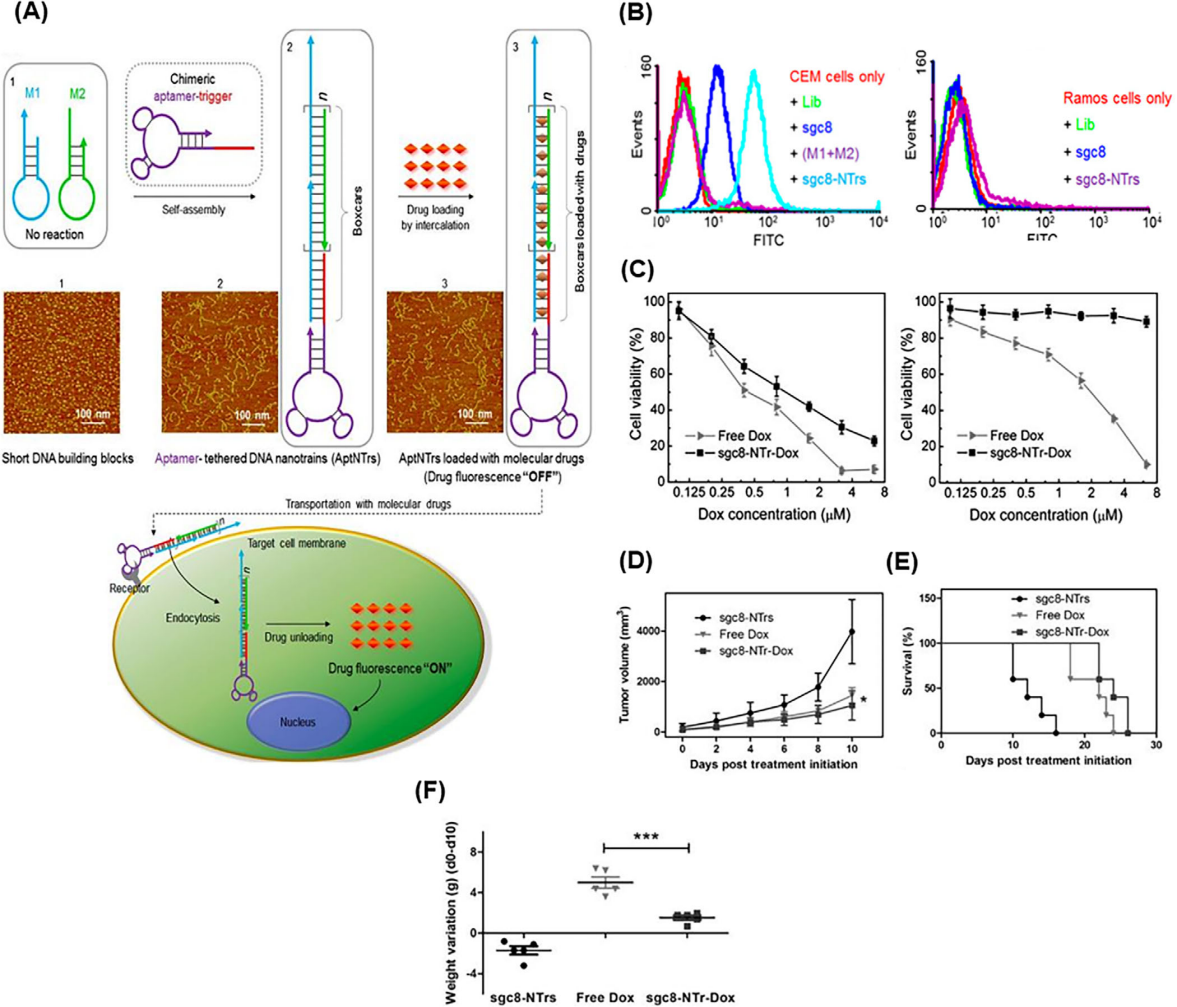
Design and function of sgc8‐NTr‐DOX complex. (A) The nanotrain was formed by the assembly of short DNA monomers M1 and M2 via cross‐hybridization induced by the sgc8 trigger. Doxorubicin (DOX) molecules were loaded on the double‐stranded regions of the nanotrain, which turned their intrinsic fluorescent off. After entering the cells, the drugs were released, which recovered their fluorescence. (B) Flow cytometry results of sgc8‐NTrs internalization in CEM cells (target) and Ramus cells (non‐target). (C) Cytotoxicity of sgc8‐NTr‐DOX and free DOX on CEM cells (left) and Ramos cells (right). (D) Comparison of tumor volume reduction (sgc8‐NTr‐DOX > free DOX > sgc8‐NTr). (E) Survival (sgc8‐NTr‐DOX > free DOX > sgc8‐NTr), and (F) weight changes (sgc8‐NTr > free DOX > sgc8‐NTr‐DOX) in mice treated by these three groups. Reprinted by permission from ref., 128 Copyright 2013 National Academy of Sciences.

For the first time in 2013, Zhu et al.^128^ designed a theranostic apt‐nanotrain for drug transport with a limited MTD. The nanotrain of sgc8 aptamer (PTK‐7 specific) engineered with a trigger probe was added to a mixture of M1 and M2 building blocks. Through the HCR reaction, the self‐assembly of nanotrain DNAs onto sgc8 aptamer as the DOX loading site was formed. The intrinsic fluorescence of DOX was effectively quenched with anchoring in the double‐stranded regions. The sgc8‐NTr‐DOX carriers entered CEM cells, human T‐cell acute lymphocytic leukemia, through PTK‐7 via endocytosis. With the release of DOX in the cell, its inherent fluorescence was restored; and by analyzing the production signals, the behavior and correct targeting of the nanotrain could be provided (Figure 24A). Flow cytometry results confirmed the selective binding ability of sgc8‐NTrs to the target cells (CEM). Multiple monomers containing DOX present in sgc8‐NTrs led to the signal amplification (Figure 24B). The degree of targeting and dose‐dependent cytotoxicity of sgc8‐NTr‐DOX in the target cells was comparable to that of free DOX. However, the toxicity and side effects in the non‐target cells (Ramos cells) were much less than that of free DOX (Figure 24C). After treating mice with sgc8‐NTr‐DOX, sgc8‐NTr without DOX, and free DOX, sgc8‐NTr‐DOX caused a decrease in tumor volume and longer survival of mice with minimal weight changes and side effects, compared to the other two groups (Figure 24D‒F). This platform with good targeting, biocompatibility, low side toxicity, and high MTDs can be promising for cancer treatment and bioimaging.

#### 3.3.2 Imaging using MBEs

**FIGURE 25:**
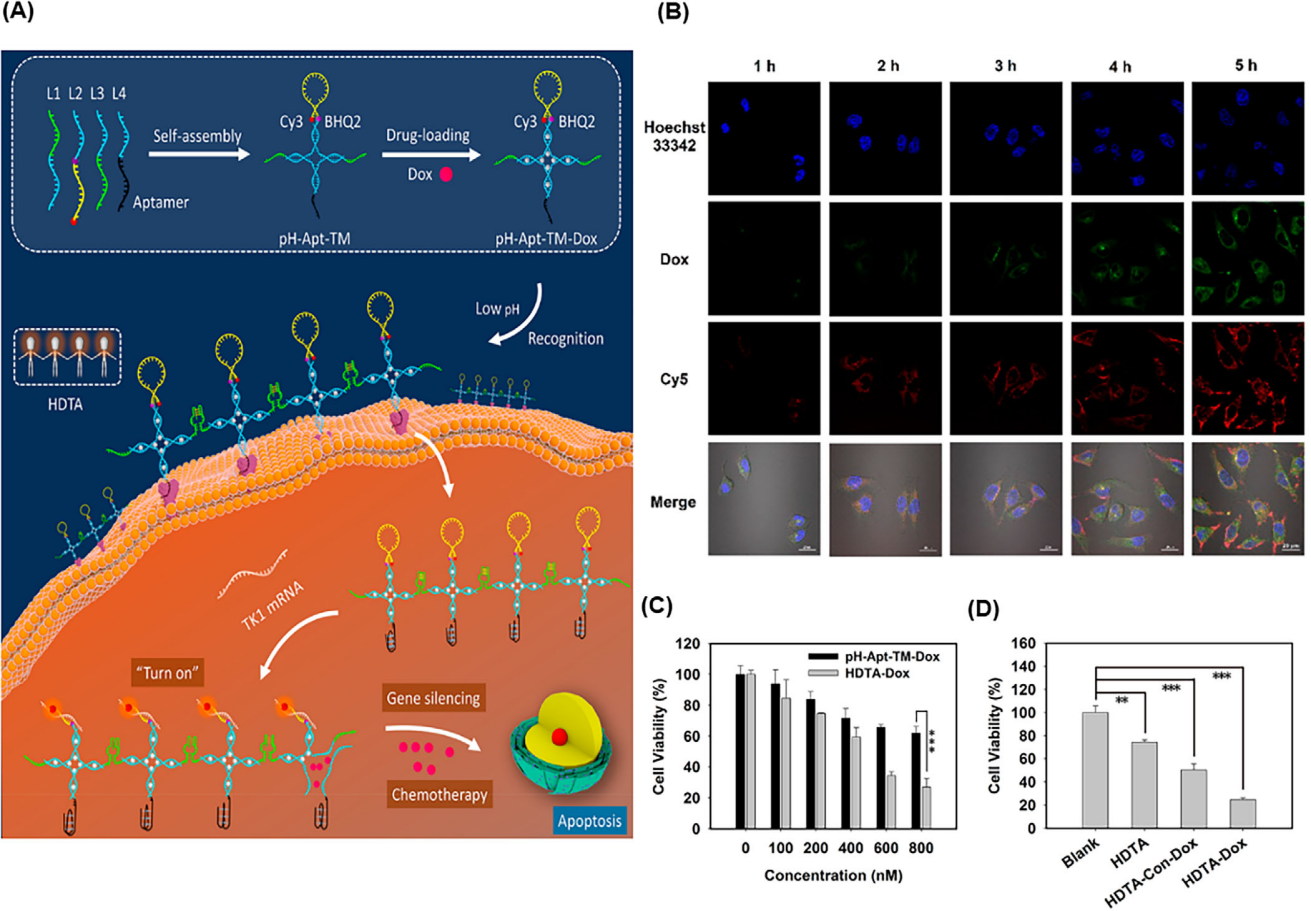
Construction steps and function mechanism of DNA‐based tile assembly of pH‒Apt‐TM‒DOX on MCF7 breast cancer cells. (A) Four single‐stranded sequences (L1, L2, L3, and L4) rich in C/G modified by AS1411 aptamer and methylene blue (MB) containing the recognition sequence of TK1 mRNA were self‐assembled. The doxorubicin (DOX) molecules were loaded on these regions. In response to the pH reduction around the cancer cell, HDTA‒DOX formed. Interacting L2 with TK1 caused gene silencing, DOX release, and finally cell apoptosis. (B) Confocal fluorescence images of the release of TK1 mRNA and DOX into MCF7 cells by hand‐in‐hand DNA tile assembly (HDTA). (C) Significant reduction of MCF7 cell viability after treatment with HDTA‒DOX compared to pH‒Apt‐TM‒DOX. (D) Comparison of cellular toxicity in the different treatments (gene‐chemo). Reprinted by permission from ref., 243 Copyright 2021 American Chemical Society.

In another study, Ma et al.^243^ introduced a novel strategy for visualization of TK1 mRNA and synergistic killing of breast cancer cells (MCF7) by using a pH‐based aptNTrs and hand‐in‐hand DNA tile assembly (HDTA). To construct a tile motif (pH‒Apt‐TM‒DOX), four CG‐rich dsDNAs were designed to load DOX, and then, it was modified by AS1411 aptamer (targeting MCF7 cells) and self‐quenched MBE containing the detection sequence of TK1 mRNA (tumor growth indicator). The low pH around cancer cells caused the accumulation of pH‒Apt‐TM‒DOX in HDTA (HDTA‒DOX) using the split i‐motif DNA, which enhanced the affinity and cell membrane permeability (Figure 25A). Inside target cells, the MBE loop was hybridized with TK1 mRNA that opened its structure, restored the CY3 fluorescence, and released DOX (Figure 25B). At the same concentration, the inhibitory effect of HDTA‒DOX on the growth of cancer cells was much higher than that of pH‒Apt‐TM‒DOX (Figure 25C). The cytotoxicity level of Apt‐TM‒DOX in the synergism of gene therapy and chemotherapy was higher than that for each component (Figure 25D). The DNA‐based tile assembly of pH‒Apt‐TM‒DOX provides significant advantages, including efficient imaging capability and a synergistic killing effect, thereby improving treatment efficacy.

#### 3.3.3 SPECT imaging

**FIGURE 26:**
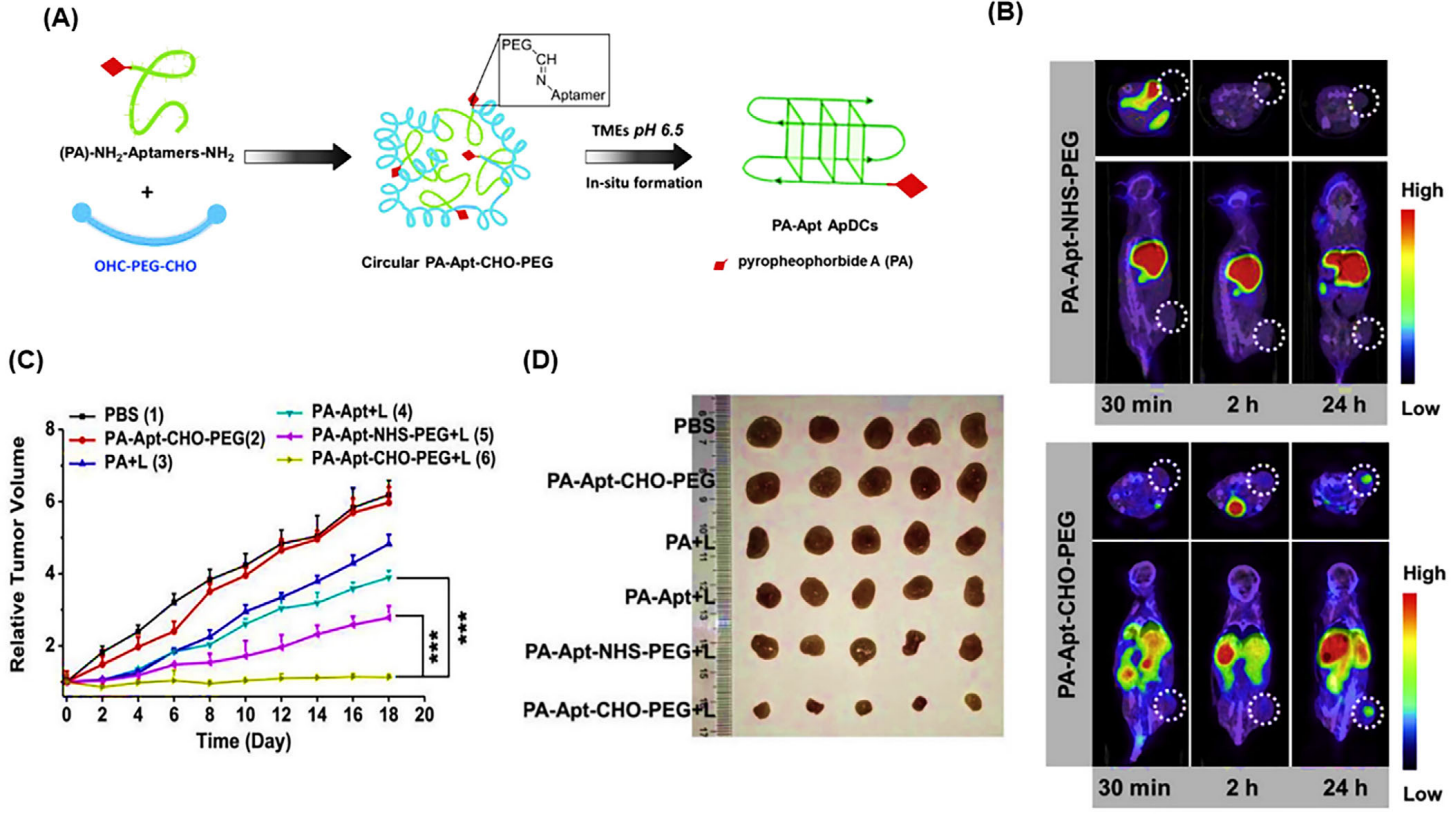
Fabrication steps and performance of PA‐Apt‒CHO‒PEG. (A) AS1411 aptamer was modified at the 3′‐ and 5′‐ends with an amine group and near its 5′‐end with a thiol group. Pyrochlorophyll A (PA) was attached to CHO‒PEG‒CHO with a covalent bond. At acidic pH, the Schiff bases were cleaved, causing the nanostructure to degrade into PA‐Apt or aptamer‐drugs (ApDCs). (B) Results of in vivo single‐photon emission computed tomography (SPECT) imaging of tumor‐bearing mice after injection of PA‐Apt‒CHO‒PEG and PA‐Apt‒NHS‒PEG. (C) Comparing the effect of the different nanostructures on tumor volume, (D) and tumor size. Reprinted by permission from ref., 244 Copyright 2020 Elsevier. CHO, Chinese hamster ovary.

Yang et al.^244^ introduced a pH‐responsive circular pyrochlorophyll A (PA) nanostructure (PA‐Apt‒CHO‒PEG) for in vivo bioimaging and improved PDT efficiency. The AS1411 aptamer (nucleolin specific) modified by dual‐amino and thiol was covalently linked to PA as a photosensitizer. Then, AS1411 was conjugated with bifunctional PEG aldehyde (CHO‒PEG‒CHO) to form the nanostructure through Schiff bases (Figure 26A). Through the utilization of γ‐rays radiation emitted by ^99m^Tc‐labeled PA and in vivo SPECT imaging, the nanostructure's tracking unveiled that PEG induced a concealed demeanor as well as prolonged retention within the bloodstream. This nanostructure decomposed into PA‐Apt in the TME (pH 6.5), which caused the targeted delivery of PA to cancer cells. Under in vitro conditions, the incubation of human breast cancer cells (MCF7) with PA‐Apt‒CHO‒PEG at pH 6.5 resulted in an increasing improvement in phototoxicity. Also, in vivo SPECT imaging (Figure 26B) was performed on mice bearing tumors (MCF7 cell line) after intravenous injection of PA‐Apt‒CHO‒PEG, and a strong gamma signal was observed at the tumor site in a time‐dependent manner. In contrast, no obvious gamma signal was observed in the control group treated with PA‐Apt‒NHS‒PEG. After treating these mice with PA‐Apt‒CHO‒PEG + light irradiation (670 nm), the biggest reduction in tumor volume and size was observed compared to other groups (Figure 26C,D). PA‐Apt‒CHO‒PEG can be a promising candidate for improving photodynamic‐based treatments in solid tumors with acidic TME.

## 4 CONCLUSION AND PERSPECTIVES

Here, we have presented a comprehensive overview of the latest advancements in the different categories of aptamer‐designed nanostructures. These structures in the forms of nanotrain, nanosponge, nanocapsule, NW, bivalent, Y‐shaped or TWJ, tetrahedron, polyhedron, and triangular origami configurations hold immense potential for tumor therapy through efficient drug carrying, targeted drug delivery, and bioimaging capabilities. As short functional nucleic acids, aptamers are crucial and pivotal in tumor therapeutic methodologies. Key attributes, such as great stability, high target affinity, low immunogenicity, ease of chemical modification, and rapid tissue penetration, serve aptamers as powerful agents for developing targeted drug delivery tools. As a result of the presented overview, aptamers have the potential to be used in the creation of multivalent tethered nanostructures (aptNTrs). These nanoconstructs incorporate several aptamers or oligonucleotides within one another, resulting in an increased affinity for targeting cancer cells and a greater number of sites for drug loading. This is made possible by the long linear structure of aptNTrs, which significantly improves therapeutic outcomes. Additionally, Cb‐Apts with the bivalent aptamer nanostructure are well‐organized for targeted drug delivery based on their significant recognition ability and molecular binding affinity. Their thermal and physical stability, high half‐life, and proper blood circulation time preferred nanotrain and bivalent aptamer‐based structures as promising agents for therapeutic aims. The available studies^78^, ^175^ clarify that DNA NWs with high drug loading capability, high aspect ratio, and tunable structure are efficient theranostics agents for cancer treatment and drug delivery. DNA TWJs by providing numerous intercalation sites for anticancer drugs, antisense oligonucleotides, and bioimaging components are also potent agents for cancer diagnostics and treatment. Based on the study by Taghdisi et al.,^188^ DNA TWJ nanostructure could be an efficient inhibitor for tumor cell growth by supplying a high loading capacity of DOX (a 1:2.5 mol ratio). Besides, aptamers in combination with triangular, tetrahedral, and polyhedral DNA origami nanostructures represent smart diagnostic, therapeutic, and bioimaging systems. So that, up to 90% suppression of tumor growth could be achieved by a triangular DNA nanostructure.^211^ Besides, recent studies^182^, ^235^ prove that tetrahedral DNA origami nanostructures with good targeting capability and antitumor function are promising scaffolds for cancer therapy. As promising frameworks, nanosponge and nanocapsule integrated aptamer nanostructures provide great efficacy for cancer treatment by offering high loading capacity for anticancer drugs. For instance, an RCA‐mediated nanosponge DNA structure can achieve a loading capacity of 5 mmol/g for DOX.^185^ Table 1 gives a summary of the available aptamer‐based nanostructures in different forms efficient in cancer therapy, gene therapy, and bioimaging. According to Table 1, aptNTrs and Cb‐Apts raise the concentration of anticancer drugs at the cancer cell site, improve their internalization into the target cells, and reduce tumor cell viability with no or less entry into non‐target cells. Correspondingly, they provide a high capacity for loading a combination of chemotherapy‒phototherapy drugs to effectively overcome multidrug resistance. Hence, aptNTrs and Cb‐Apts as the conceptual frameworks and efficient toolboxes are ideal for developing novel targeted cancer therapy. In addition to aptNTrs, triangular and tetrahedral aptamer nanostructures are promising skeletons for cancer chemo‐phototherapy by providing a high loading capacity of anticancer drugs and thermal therapy agents (Table 1). Also, the integration of aptamers into bivalent nanostructures yields robust instruments for bioimaging purposes, owing to their exceptional biocompatibility, convenient modifiability, and capacity for size manipulation. Based on Table 1, aptNTr, TDN, and Y‐shaped nanostructures have been utilized as efficient gene therapy agents. Hence, there is great potential to apply other types of aptamer‐tethered nanostructures to identify those with the most gene therapy efficacy for future use. The available developments toward the application of aptamer‐integrated nanostructures in the different forms of nanosponge, NW, nanocapsule, bivalent, Y‐shaped, triangular, tetrahedron, and polyhedron origami for cancer cure highlight that the study of will open new perspectives for meeting the needs of health care and therapeutics.

**TABLE 1:** A summary of the aptamer‐based nanostructures in the various forms functional in the different biomedical fields.

Applications	Delivery systems	Materials	Therapeutics agents	Drug loading (nanostructure:drug)	Targets	Advantages	Ref.
**Drug delivery**							
Chemotherapy	NWs‐aptamer‐DOX	Aptamer Sgc8, DNA nanowire	DOX	Each structural unit loads 64 DOX molecules	Human cervical carcinoma cells (HeLa)	Nuclease resistance, high drug loading capacity, biocompatible, and minimal side effects	^175^
	AS1411NTrs	AS1411 (trigger modified) and monomeric hairpin sequence H1 and H2	DAU, EPI, and DOX	1:5 molar ratio (AS1411NTrs:DAU), 1:5 molar ratio (AS1411NTrs:EPI), and 1:5 molar ratio (AS1411NTrs:DOX)	Human cervical cancer cells (HeLa)	High ability to load different drugs, binding and targeted drug delivery, and low drug toxicity on healthy cells	^176^
	AS1411NTrs	AS1411 aptamer (trigger modified), monomeric hairpin sequence H1/H2, and divalent ions (Mg^2+^, Zn^2+^, or Mn^2+^)	Mithramycin	1:2 molar ratio	Human liver cancer cells (HepG2)	Improvement drug binding	^177^
	6N‐LZH5B‐NTr‐Dox	6N‐Apt‐trigger and monomeric hairpin sequence (6NM1‒6NM2)	DOX	1:50 molar ratio	Human liver cancer cells (HepG2)	Stability, selective cell recognition, and non‐targeted non‐toxicity	^178^
	NT8	Aptamer (trigger modified) and monomeric hairpin sequence H1 and H2	DOX	50:1 molar ratio	Human pancreatic ductal adenocarcinoma cells (PL45)	Significant targeting and cytotoxicity to cancer cells with minimal side effects on healthy cells	^179^
	B1 NT‐EP	B1 aptamer (trigger modified) and monomeric hairpin sequence M1 and M2	EPI	1:160 molar ratio	Human bladder cancer cells (T24 and KU‐7)	High targeting capability and selective toxicity	^180^
	Doxo@Apt‐DNA‐icosa	Five‐point‐star shape ssDNA and MUC1 aptamer	DOX	Each structural unit loads 1200 DOX molecules	Human breast cancer cells (MCF7)	High selectivity, function as nanocages, high rigidity, and unaffected by deformation	^181^
	HApt‐tFNA@Dxd	HER2 aptamer	Dxd	1:3 molar ratio	Human Gastric cancer cells (HGC‐27 and NCI‐N87)	Low cost, high affinity to target cells, and high loading capacity	^182^
	Cb‐Apt‐Dox	Two AS1411 aptamers	DOX	1:5 molar ratio	Breast cancer cells (MCF7) and mouse breast cancer cells (4T1)	Significant killing effect on target cells, few effects on healthy cells, and resistance to digestion by nucleases	^183^
	DOX@RCA‐ZnO‐NSs	DNAzyme NSs, sgc‐8c aptamers, ZnO NPs	DOX	1:5 mmol/g	Human cervical carcinoma cells (HeLa)	Multivalent delivery, resilience against nuclease degradation, and selective targeting	^185^
	TWJ pocket with DOX	Three strands of AS1411 aptamer	DOX	1:2.5 molar ratio	Human prostate cancer cells (PC‐3), mouse breast cancer cells (4T1)	Strong stability, pH‐sensitive controlled release, specific targeting of tumor cells, and minimal side effect	^188^
Combination therapy	AS1411NTrs + TBO + DOX	AS1411 aptamer (trigger modified) and monomeric hairpin sequence H1 and H2	DOX and TBO	1:2:3 molar ratio (AS1411NTrs:TBO:DOX)	Human breast cancer cells (MCF7 and ADR)	Overcoming drug resistance, improvement of the treatment process, and an increase in the inhibition of target cells	^205^
	TA6NT‐AKTin‐DOX	TA6 aptamer (trigger modified) and monomeric hairpin sequence M1 and M2 (conjugated with AKTin)	DOX	1:20 molar ratio	Breast cancer stem cells	Ability to protect the drug against enzymatic degradation, high ability in targeted drug delivery, and less toxicity for healthy cells	^206^
	XQ‐2d‐NT‐PTX/CA4	XQ‐2d aptamer (trigger modified) and monomeric hairpin sequence H1 and H2	PTX and CA4	1:5:5 molar ratio (XQ‐2d‐NT:PTX:CA4)	CD71^+^	Synergistic therapeutic effect and ability to reset for most of the required drugs	^207^
	ACT@DM + L	AS1411 aptamer and CpG	DOX and MB	‒	Mouse breast cancer cells (4T1)	Precisely targeting tumor cells, boost cellular internalization, and exerting anticancer properties	^210^
	TOADI + laser	M13mp18 ssDNA, AS1411 aptamer, and ICG	DOX and ICG	0.4 nM:20 µM:14 µM (DNA origami:DOX:ICG)	Mouse breast cancer cells (4T1)	Stability, high biocompatibility, and superiority in the transport of multiple oligonucleotides	^211^
Chemodynamic therapy	aptNTDNA‐CuNCs	S9C8 aptamer (trigger modified), CuNCs, and monomeric hairpin sequence H1 and H2	‒	‒	Human cervical carcinoma cells (HeLa)	Improving CDT, biocompatible, targeting cancer cells and the least damage to healthy cells	^212^
Cytotoxic proteins	Cb‐Apt‐*β*CD	Two sgc8 aptamer (modified), *β*CD, AdA	Saporin protein	1:50 molar ratio (TMR‐apt‐*β*CD:AdA)	Human cervical carcinoma cells (HeLa)	Enhanced intracellular protein delivery	^214^
**Oligonucleotide therapy**							
Gene therapy	siSTAT3‐DOX‐Assembly	Three short strands (siRNA complementary, aptamer sequence, and complementary sequence to load DOX)	DOX and siRNA	Concentration ratio 1:50 (RCA:siRNA) and about 200 DOX molecules	Mouse melanoma cells (B16)	Dual delivery of chemotherapy and genetic drugs	^232^
	ApTL/mEGFP/DOX	Liposome, AS1411 aptamer, mRNA, and plasmid	DOX, mRNA, and plasmid	1:20 ratio	Human breast cancer cells (MDA‐MB‐231) and human cervical carcinoma cells (HeLa)	High loading capacity, long‐term blood circulation, safe, and specific targeting	^233^
	TY1Y2 (siBcl2/siSurvivin)	EpCAM‐aptamer and two Y‐shaped DNA	siBcl2, siSurvivin, and DOX	‒	Human hepatocellular carcinoma cells (Huh‐7)	Decreasing off‐target impacts, facilitating cellular internalization, biostability, and resistance to DNase I	^235^
**Bioimaging**							
Imaging using anthracyclines	Ce6‐DOX‐DNA	Three types of single‐strained DNA (functionalized by TLS11a, Ce6, and RQ)	DOX and Ce6	‒	Hepatocellular carcinoma (HepG2)	Adjustable switch “ON” capability and combining chemotherapy, PDT, and real‐time NIR fluorescence imaging	^242^
	sgc8‐NTr‐DOX	Sgc8 (trigger modified) and monomeric hairpin sequence M1 and M2	DOX	1:50 molar ratio	Human T‐cell acute lymphocytic leukemia cells (CEM)	Drug transport with limited maximum tolerated dose, high ability in targeting, killing target cells, and along with imaging	^128^
Imaging using MBE	pH‐Apt‐TM‐DOX	AS1411 aptamer, molecular beacon, TK1 mRNA, and monomeric hairpin sequence (L1, L2, L3, and L4)	DOX	1:5 molar ratio	Human breast cancer cells (MCF7)	Improving affinity and stability of cell membrane permeability, imaging capability, and a synergistic killing effect	^243^
SPECT imaging	PA‐Apt‐CHO‐PEG	AS1411 aptamer (modified), PA, and PEG aldehyde	‒	‒	Human breast cancer cells (MCF7)	Improving PDT efficiency	^244^

Abbreviations: Cb‐Apt, circular bivalent aptamer; CDT, chemodynamic therapy; Ce6, chlorin e6; CHO, Chinese hamster ovary; DOX, doxorubicin; EPI, epirubicin; ICG, indocyanine green; MB, methylene blue; NIR, near‐infrared; PA, pyrochlorophyll A; PTX, paclitaxel; SPECT, single‐photon emission computed tomography; ssDNA, single‐stranded DNA; TBO, toluidine blue O; TWJ, three‐way junction; *β*CD, *β*‐cyclodextrin.

## AUTHOR CONTRIBUTIONS

F.M., H.Z., and F.Z. wrote the manuscript and drew the figures and tables. S.M.T., K.A., and Z.K. contributed to the design of the manuscript structure and edited the final manuscript. M.R. and M.A. assessed and reviewed the manuscript structure and ideas. All authors have read and approved the final manuscript.

## CONFLICT OF INTEREST STATEMENT

The authors declare they have no conflicts of interest.

## ETHICS STATEMENT

Not applicable.

## Data Availability

The data that support the findings of this study are openly available at https://doi.org/10.1002/mco2.775.
